# Supplementary material for: Scalable synthesis enabling multilevel bio-evaluations of natural products for discovery of lead compounds
Source: Nat Commun. 2018 Mar 29;9:1283. doi: 10.1038/s41467-018-03546-9 (PMC5876371; doi:10.1038/s41467-018-03546-9)
Supplement: Supplementary file 1 — Supplementary Information [file 41467_2018_3546_MOESM1_ESM.pdf]

Scalable Synthesis Enabling Multilevel Bio-Evaluations of Natural Products for Discovery of Lead Compounds

Zhu *et al*.

Supplementary Figures

A HL60

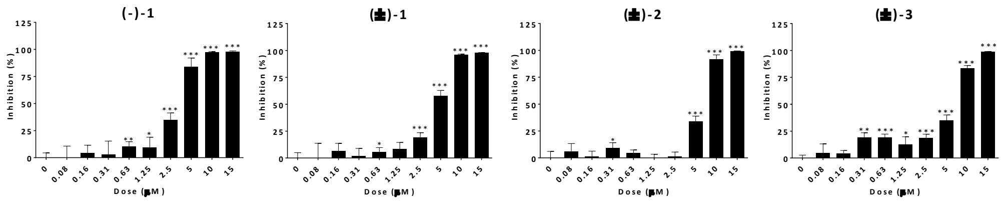

B SMMC7721

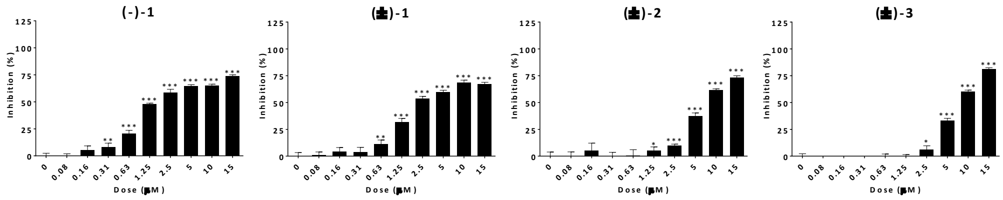

C A549

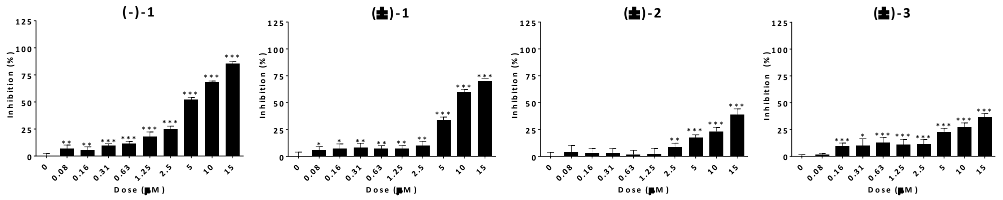

D MCF7

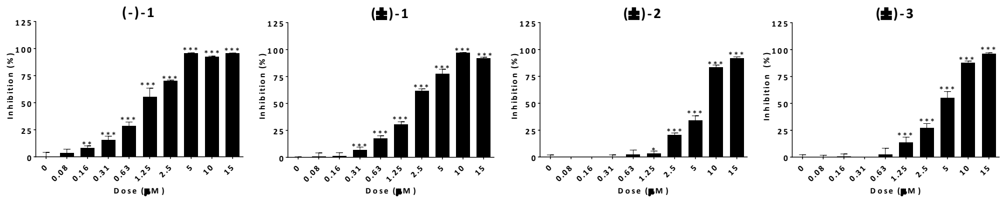

E SW480

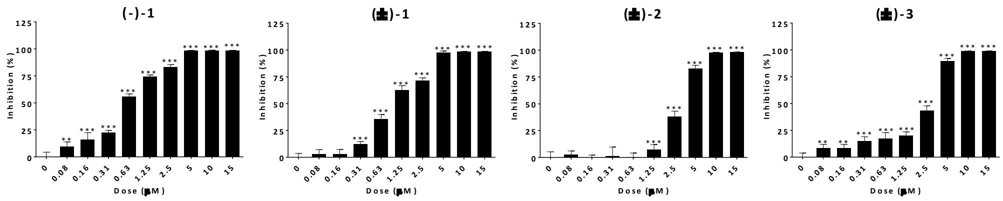

Supplementary Figure 1. Effect of *Isodon* diterpenoids on cancer cell growth Cell lines were treated with different doses of natural (-)-**1** or synthetic (±)-**1**–**3** for 48 h. Cell viability was measured by MTT assay. MTT results of (**A**) HL60, (**B**) SMMC7721, (**C**) A549, (**D**) MCF7 and (**E**) SW480 cells. Experiments were conducted in triplicate and results are shown as means of three experiments. Error bars represent ± SD. **P < 0.05*, ***P < 0.01*, ****P < 0.001* compared to control (Student's *t*-test).

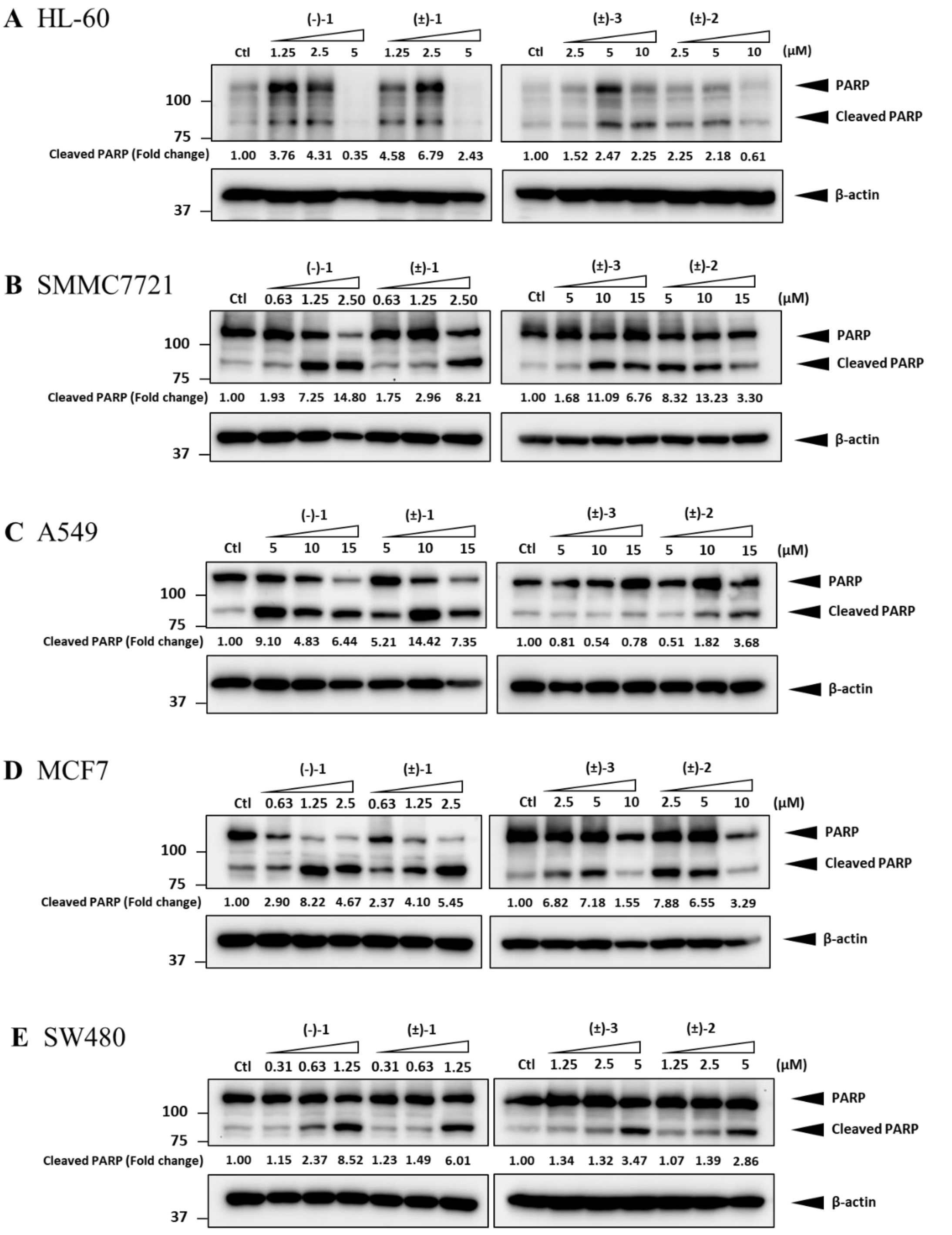

Supplementary Figure 2 Apoptotic effects of *Isodon* diterpenoids. Cells were treated with the indicated concentrations of natural (–)-**1** or synthetic (±)-**1**–**3** for 48 h. The protein levels of apoptosis marker cleaved PARP and PARP were detected by Western blotting. β-actin was used as an internal loading control. Representative immunoblots and quantifications for (**A**) HL60, (**B**) SMMC7721, (**C**) A549, (**D**) MCF7 and (**E**) SW480.

A

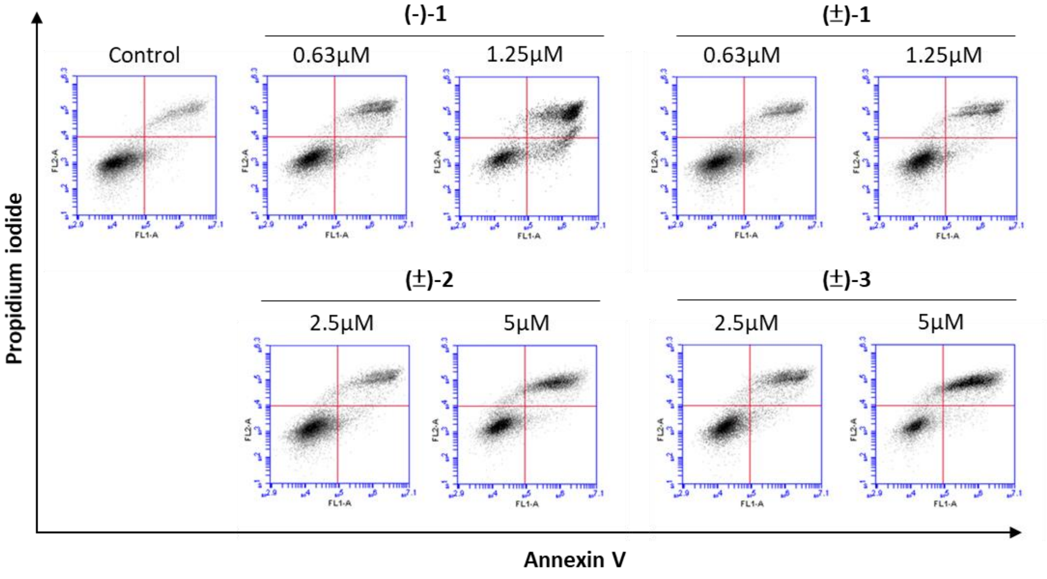

B

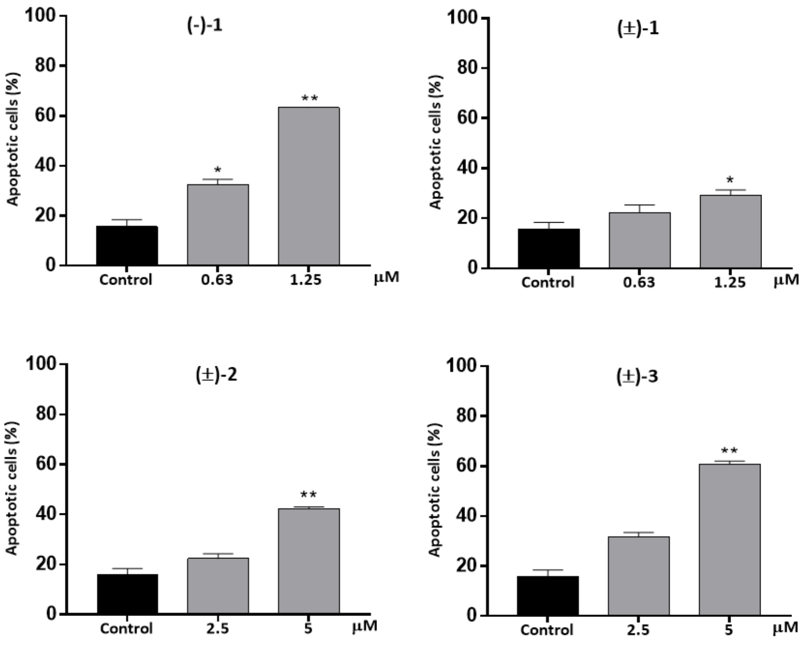

Supplementary Figure 3 Flow cytometric analysis of apoptosis. SW480 cells were treated with the indicated concentrations of (–)-**1** or (±)-**1–3** for 48 h, stained with Annexin V-Alexa Fluor 488/PI and analyzed via flow cytometry (3 × 10^4 events per sample). **(A)** Representative flow cytometric analysis results of *Isodon* diterpenoid-induced apoptosis. **(B)** Apoptotic cell percentage. Experiments were conducted in triplicate and data are shown as means of three experiments. Error bars represent ± SD. **P < 0.05; **P < 0.01* compared to control (Student's *t*-test).

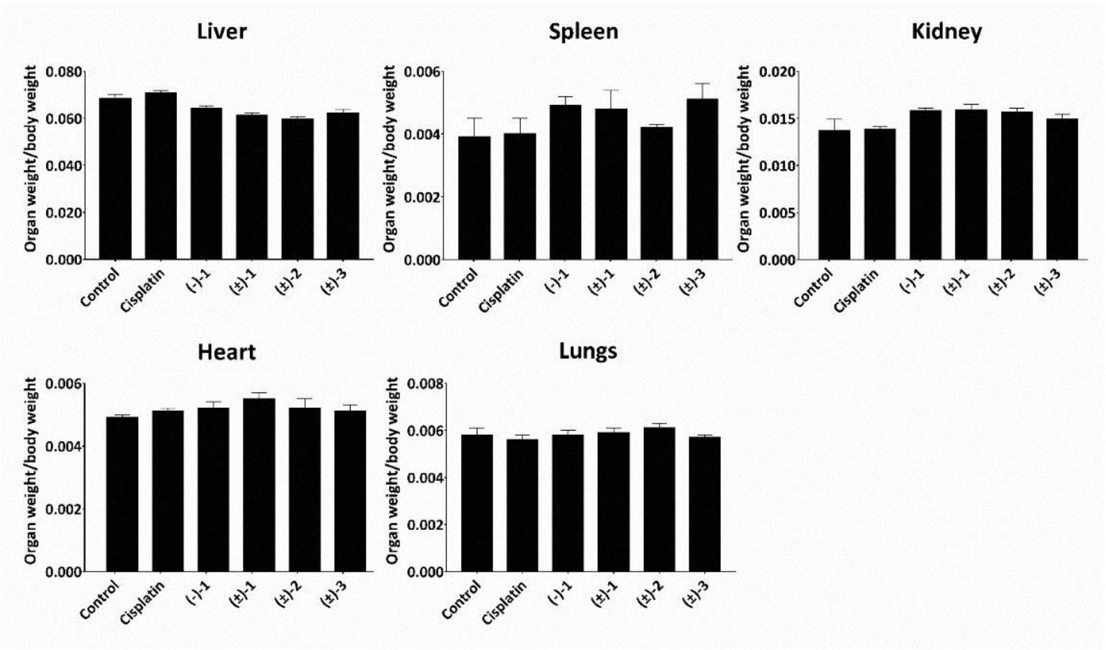

Supplementary Figure 4 ***Isodon*** **diterpenoids did not affect mouse organ weights.** After the 21 day treatment period, mice (n = 7 mice per group) were sacrificed, and organs were removed and weighed to assess potential drug toxicity. Data shown are means ± SEM of organ weights normalized to body weight for seven mice. Error bars represent ± SEM.

A

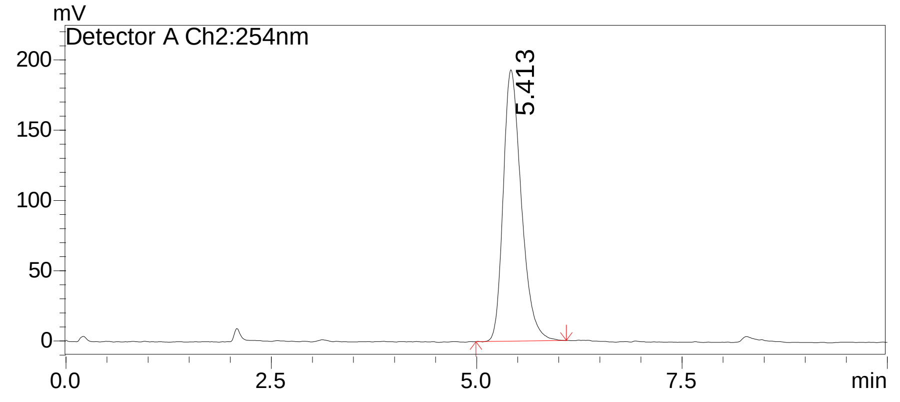

Peak No.	Time	Area	Area %	T. plate	Tailing	Resolution
1	5.413	2970696	100.000	2873.639	1.349	--

B

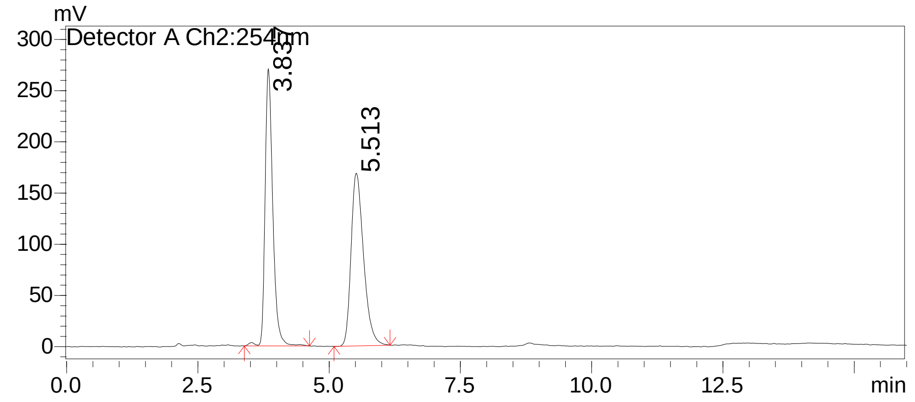

Peak No.	Time	Area	Area %	T. plate	Tailing	Resolution
1	3.838	2761406	49.6595	3327.985	1.406	--
2	5.513	2799277	50.3405	2587.075	1.375	4.789

Supplementary Figure 5 HPLC analysis. (A) natural (–)-**1** and **(B)** synthetic (±)-**1.** Conditions: chiralpak IA column (0.46 cm I.D. × 15 cm L), eluted with MeOH (100%), flow rate = 1.0 mL/min, detector = UV at 254 nm, temperature = 35 ℃.

A

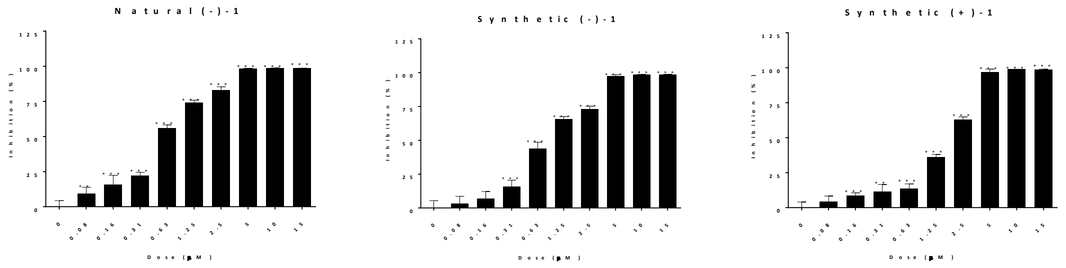

B

Compounds	SW480
Natural (–)-**1**	0.598
Synthetic (–)-**1**	0.854
Synthetic (+)-**1**	1.670

Supplementary Figure 6 *In vitro* cancer inhibition of synthetic (–)-1 and (+)-1. To assess the cancer inhibitory activity of synthetic (–)-**1** and (+)-**1**, SW480 cells were treated with different doses of natural (–)-**1**, synthetic (–)-**1** and (+)-**1** for 48 h. Cell growth inhibition was measured by MTT assay. Natural and synthetic (–)-**1** exhibited comparable levels of cancer growth inhibition. Synthetic (+)-**1** also showed significant growth inhibitory activity. (**A**) Results of MTT assay. (**B**) IC_{50} values as expressed in μM. Experiments were conducted in triplicate. For (**A**), results are shown as means of three experiments. Error bars represent ± SD. **P < 0.05, **P < 0.01, ***P < 0.001* compared to control (Student's *t*-test).

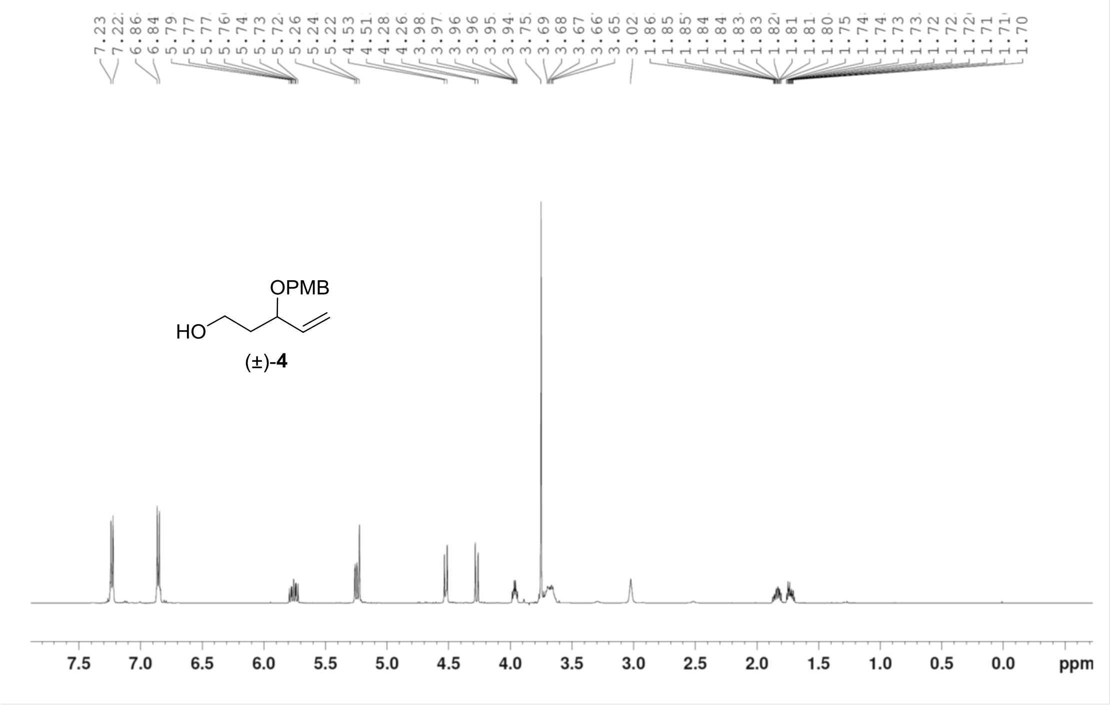

Supplementary Figure 7. ^{1}H NMR spectrum of (±)-**4** (300 MHz, $CDCl_3$)

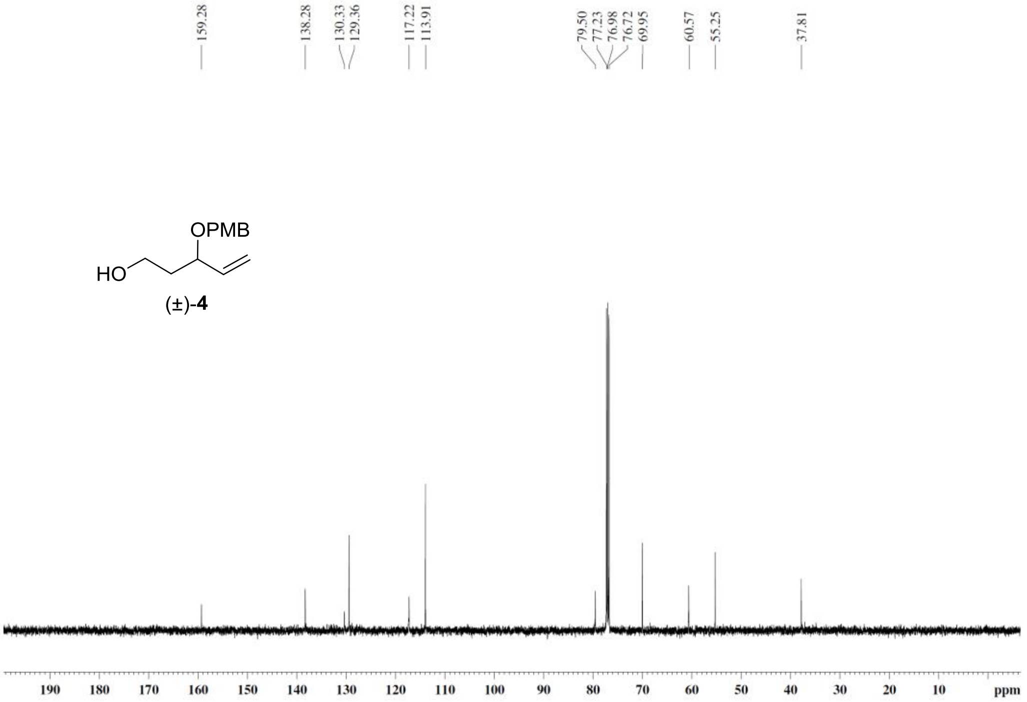

Supplementary Figure 8. ^{13}C NMR spectrum of (±)-**4** (76 MHz, $CDCl_3$)

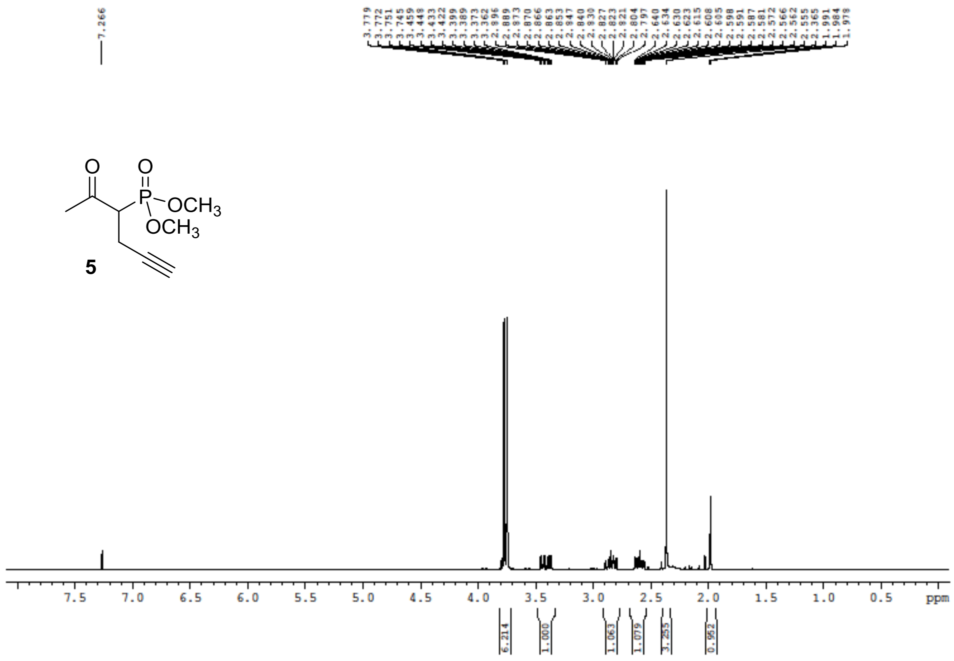

Supplementary Figure 9. ^{1}H NMR of **5** (500 MHz, $CDCl_3$)

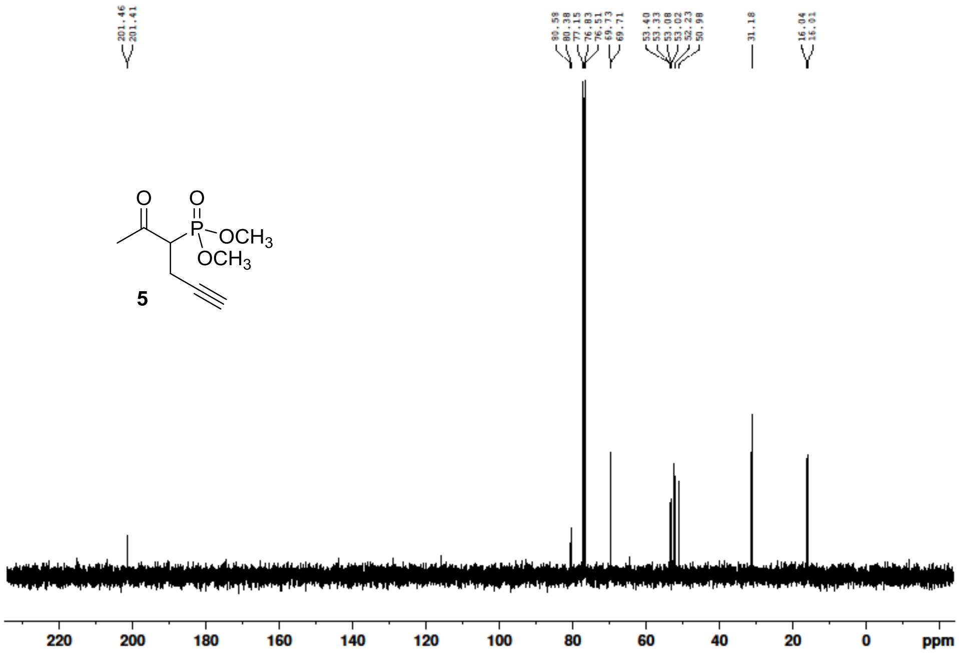

Supplementary Figure 10. ^{13}C NMR of **5** (126 MHz, $CDCl_3$)

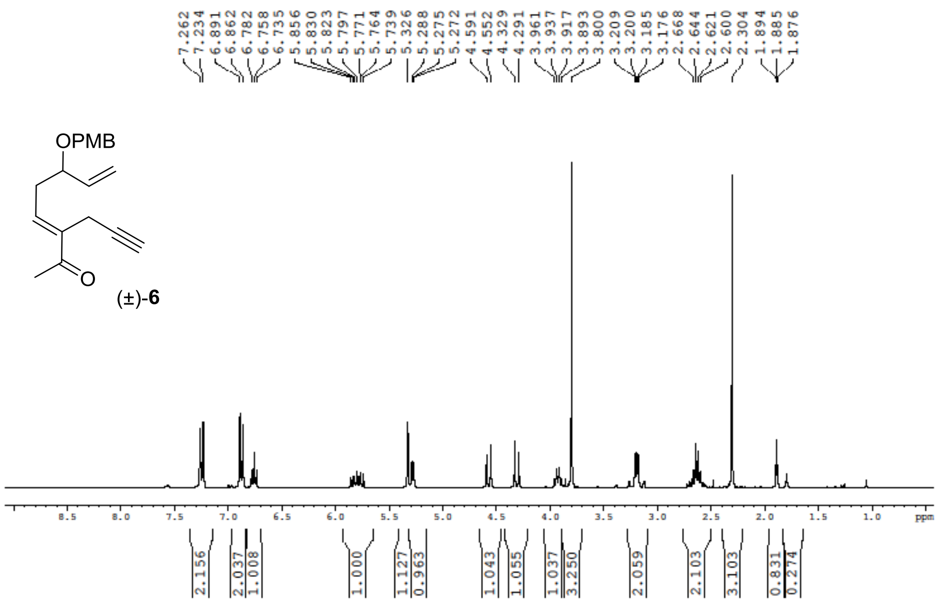

Supplementary Figure 11. ^{1}H NMR of (±)-**6** (300 MHz, $CDCl_3$)

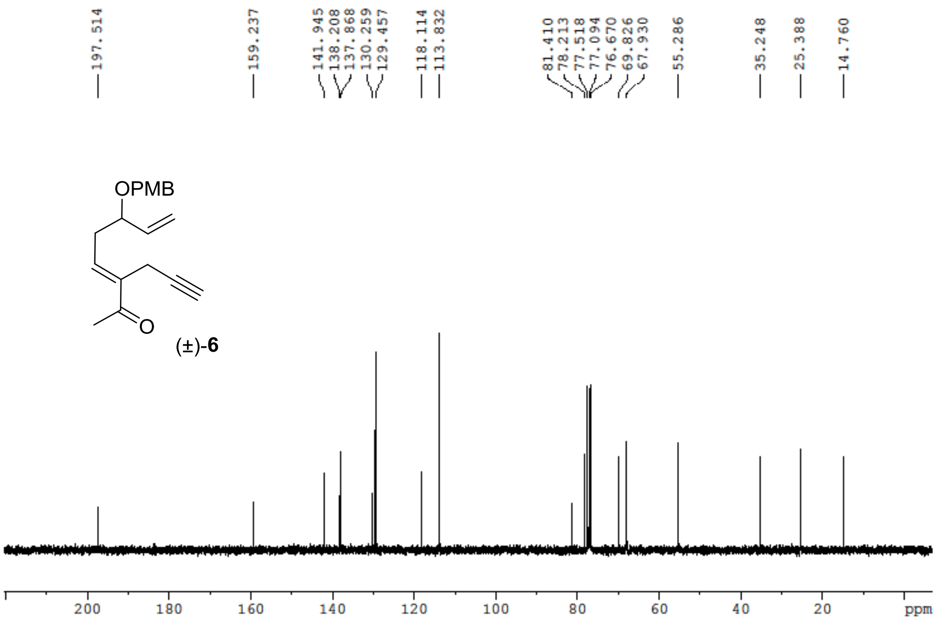

Supplementary Figure 12. ^{13}C NMR of (±)-**6** (76 MHz, $CDCl_3$)

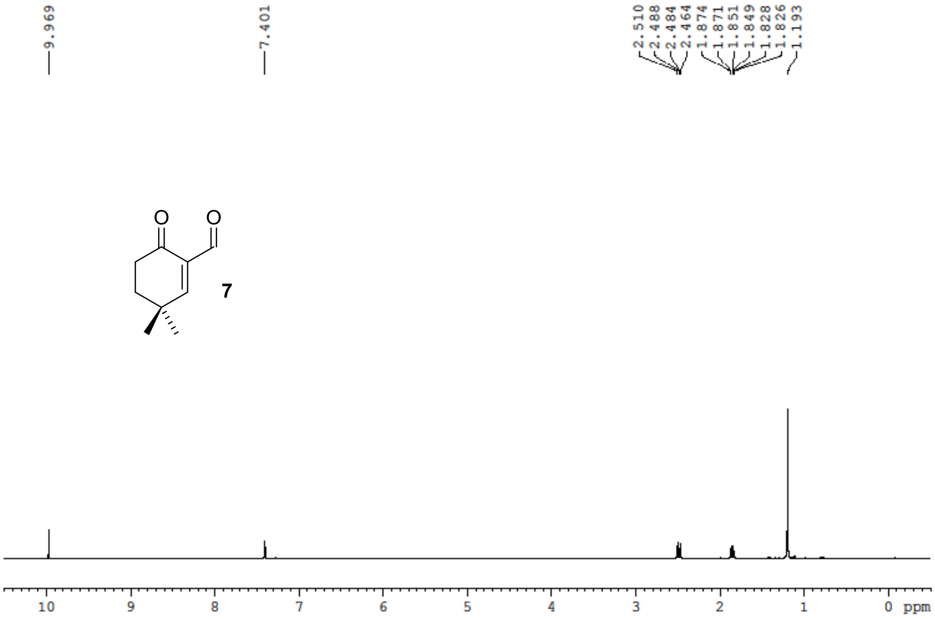

Supplementary Figure 13. ^{1}H NMR of **7** (300 MHz, $CDCl_3$)

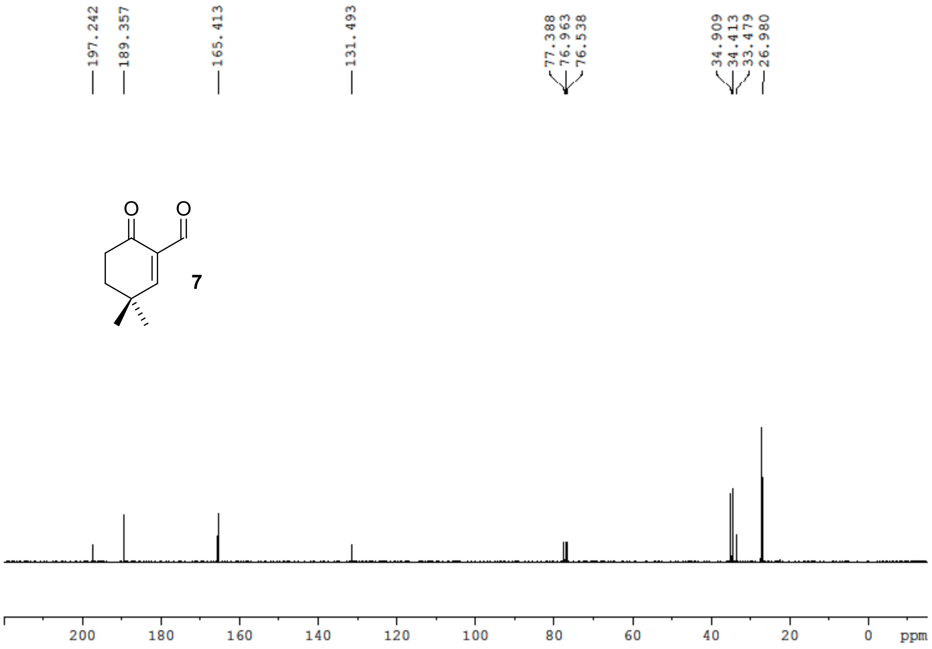

Supplementary Figure 14. ^{13}C NMR of **7** (76 MHz, $CDCl_3$)

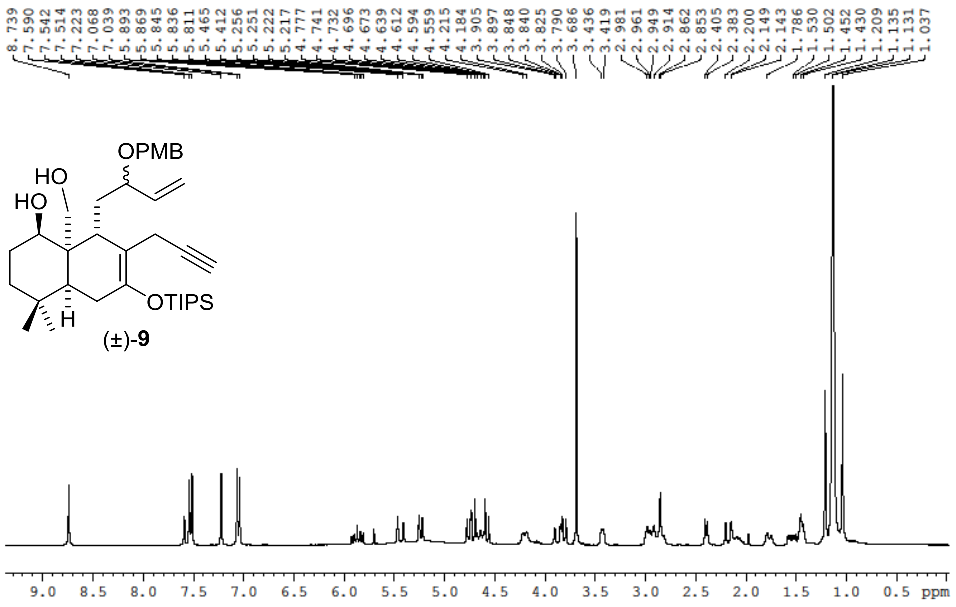

Supplementary Figure 15. ^{1}H NMR of (±)-**9** (high R_f) (300 MHz, pyridine-d_5)

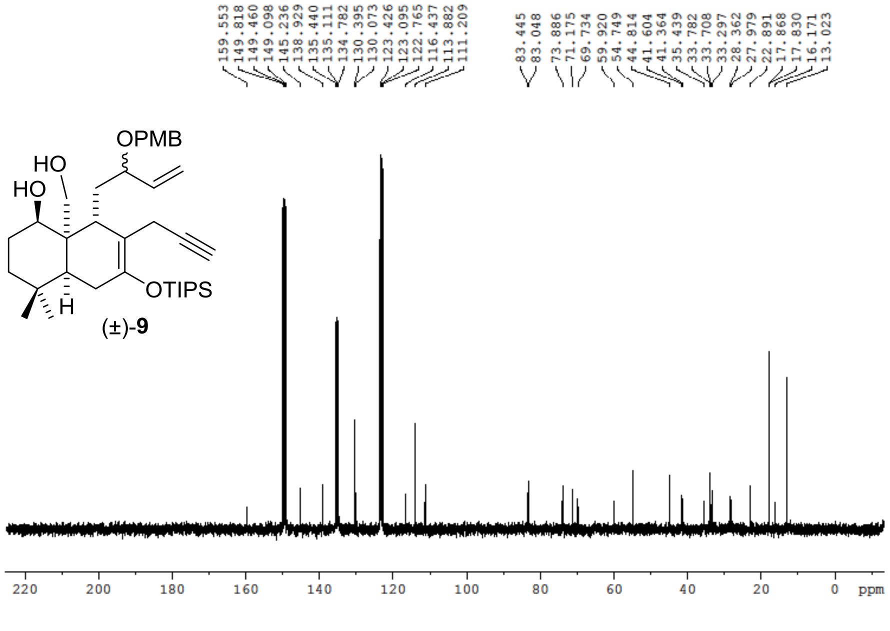

Supplementary Figure 16. ^{13}C NMR of (±)-**9** (high R_f) (76 MHz, pyridine-d_5)

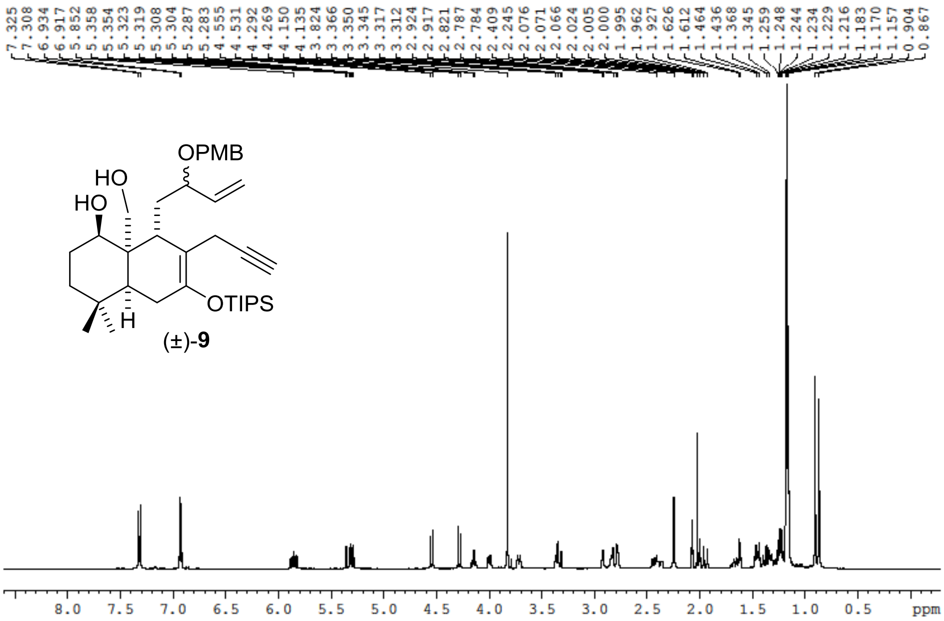

Supplementary Figure 17. ^{1}H NMR of (±)-**9** (low R_f) (500 MHz, CD_3CN)

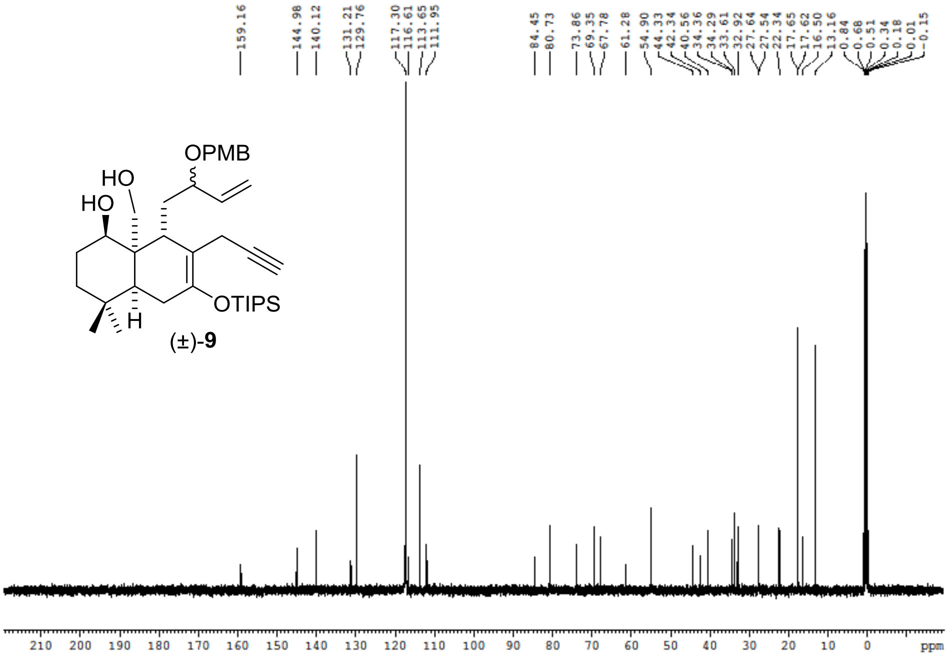

Supplementary Figure 18. ^{13}C NMR of (±)-**9** (low R_f) (126 MHz, CD_3CN)

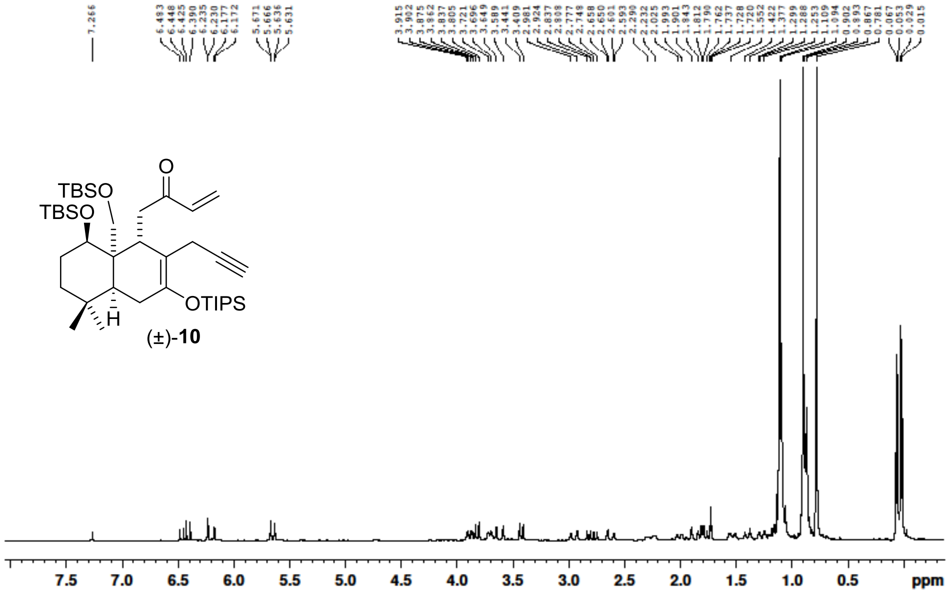

Supplementary Figure 19. ^{1}H NMR of (±)-**10** (300 MHz, $CDCl_3$)

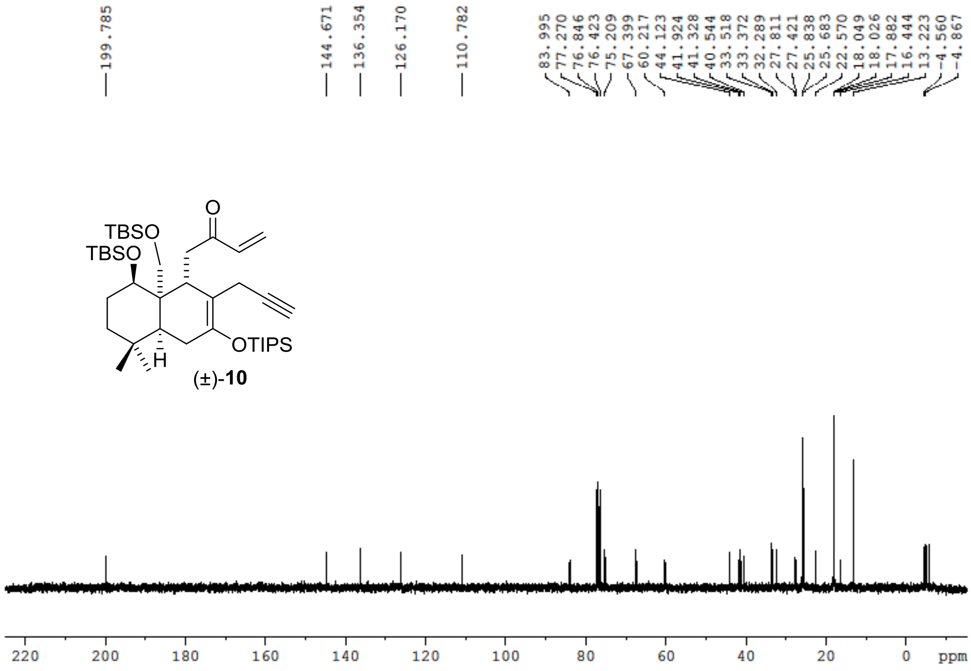

Supplementary Figure 20. ^{13}C NMR of (±)-**10** (76 MHz, $CDCl_3$)

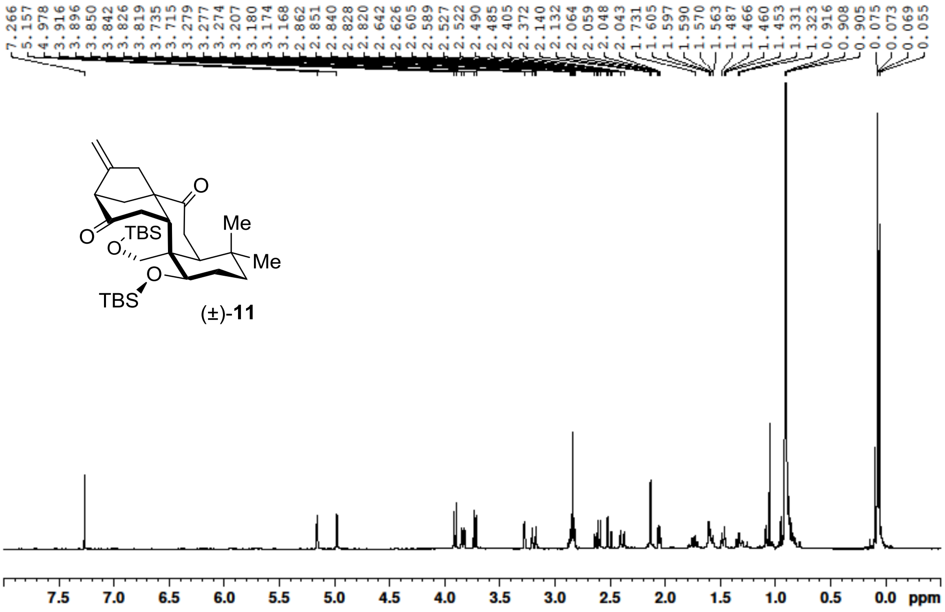

Supplementary Figure 21. ^{1}H NMR of (±)-**11** (500 MHz, $CDCl_3$)

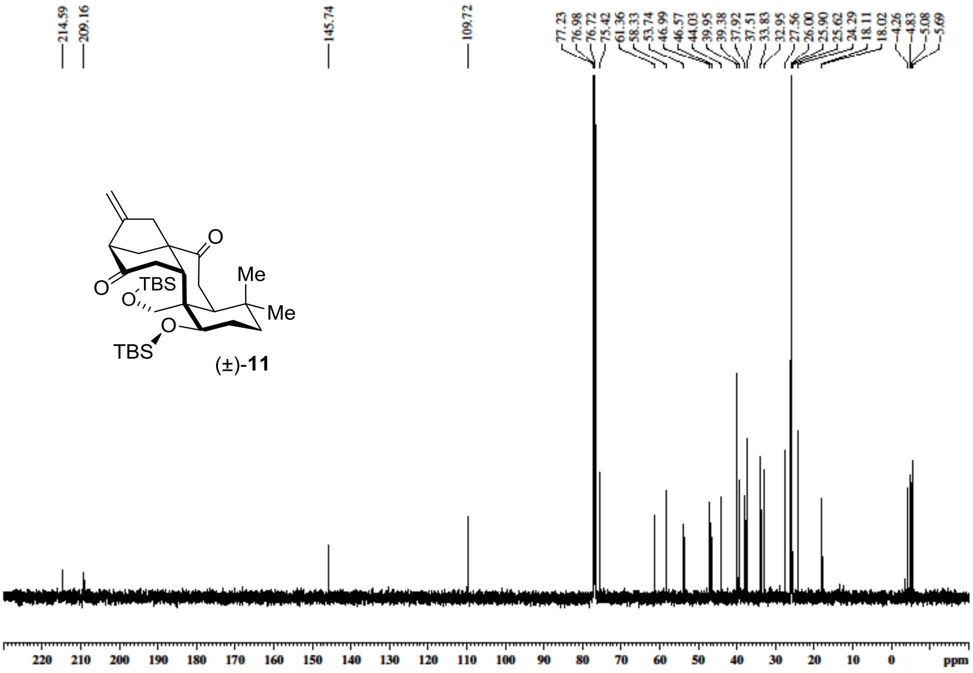

Supplementary Figure 22. ^{13}C NMR of (±)-**11** (126 MHz, $CDCl_3$)

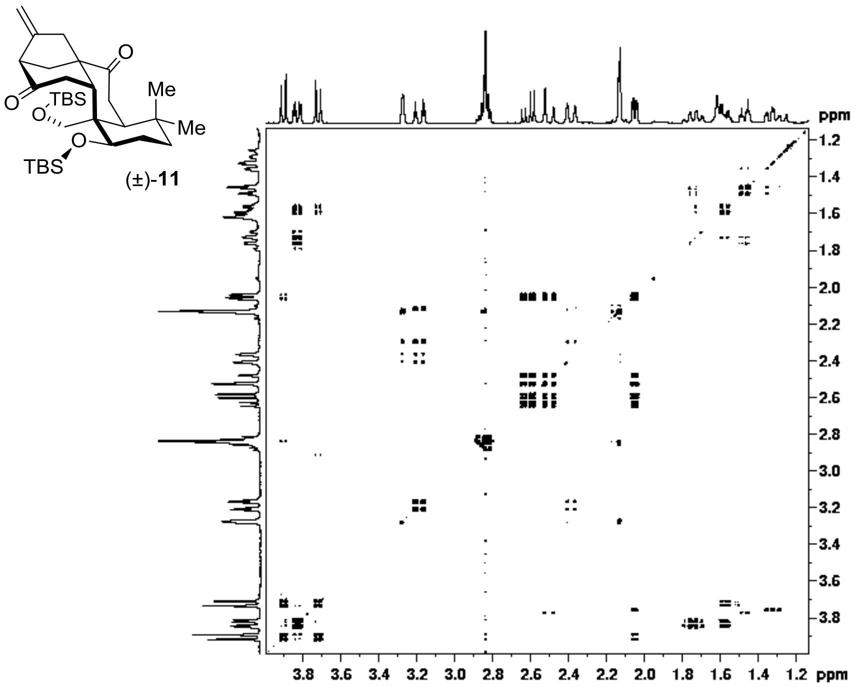

Supplementary Figure 23. COSY NMR of (±)-**11** (500 MHz, $CDCl_3$)

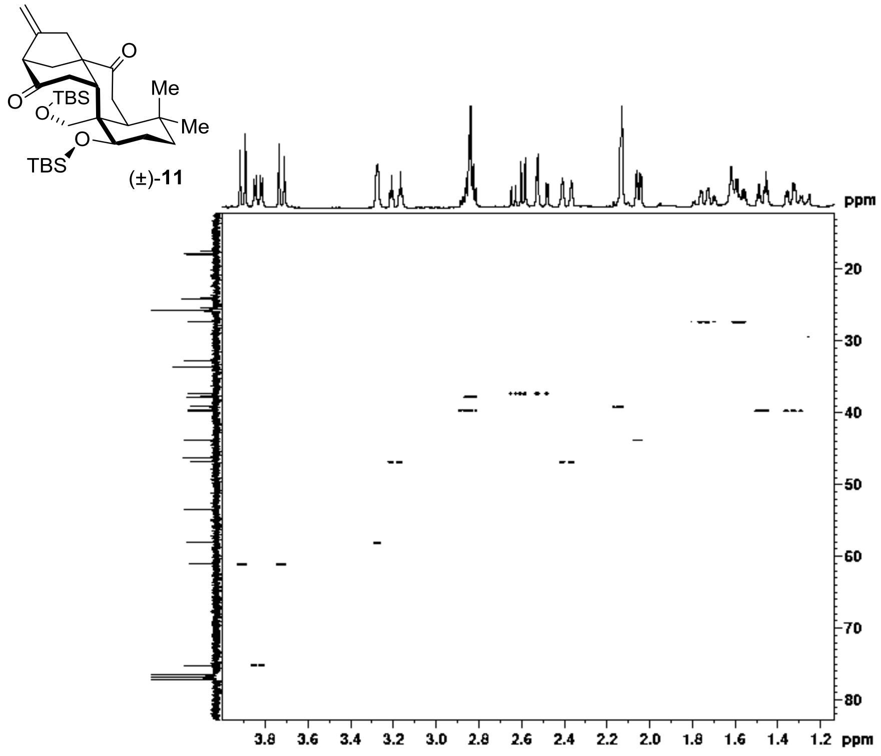

Supplementary Figure 24. HSQC NMR of (±)-**11** (500 MHz, $CDCl_3$)

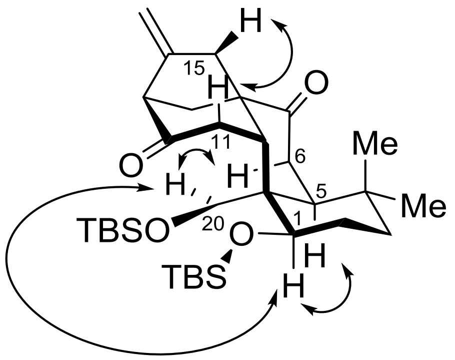

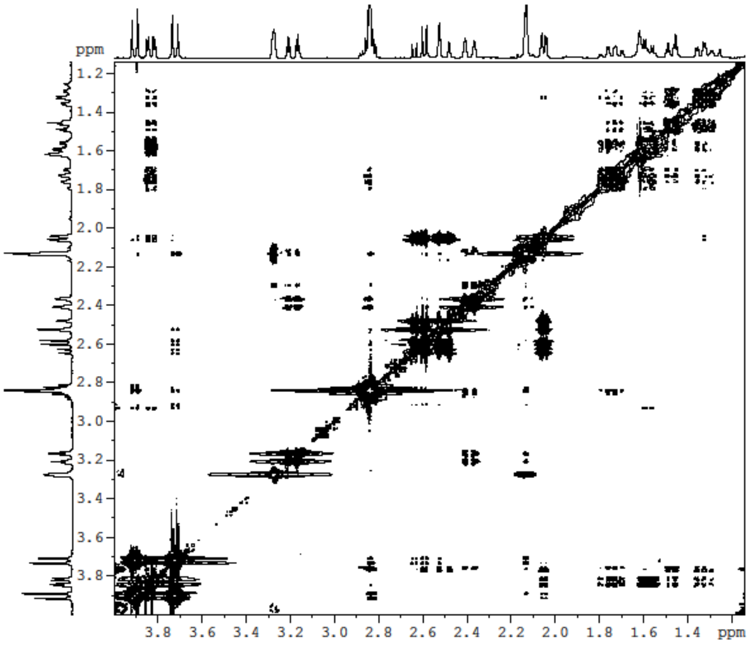

Supplementary Figure 25. NOSEY NMR of (±)-**11** (500 MHz, $CDCl_3$)

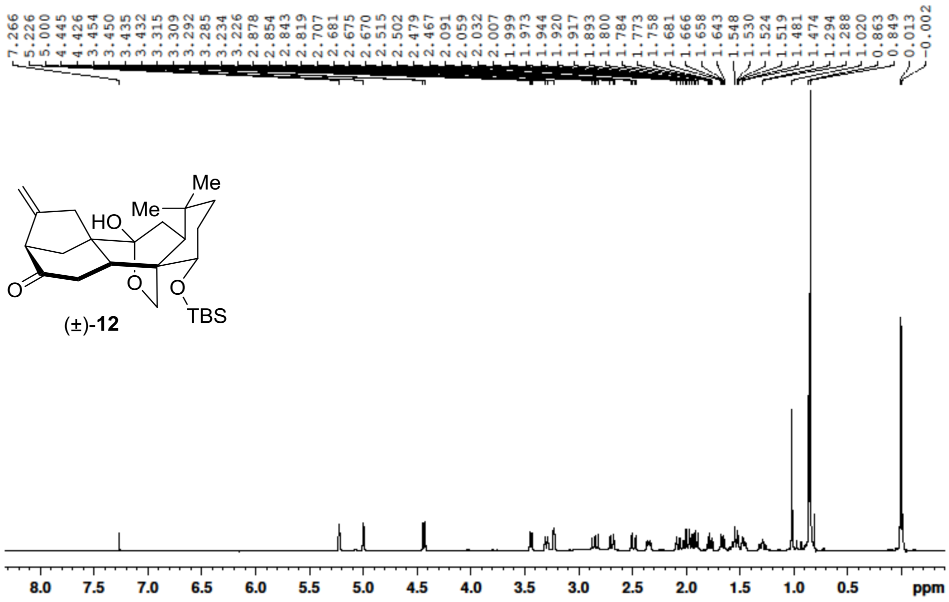

Supplementary Figure 26. ^{1}H NMR of (±)-**12** (500 MHz, $CDCl_3$)

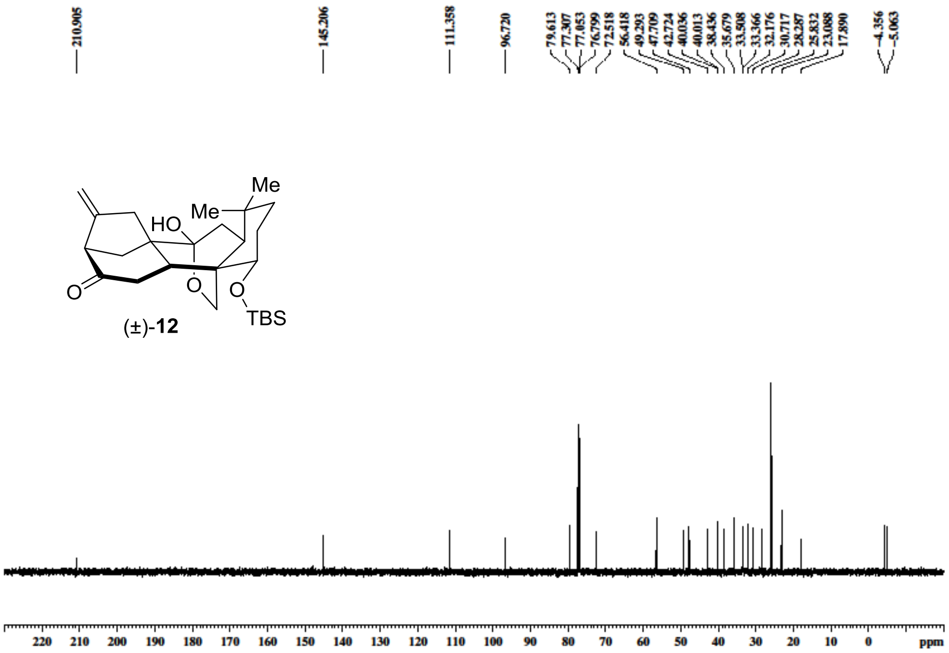

Supplementary Figure 27. ^{13}C NMR of (±)-**12** (126 MHz, $CDCl_3$)

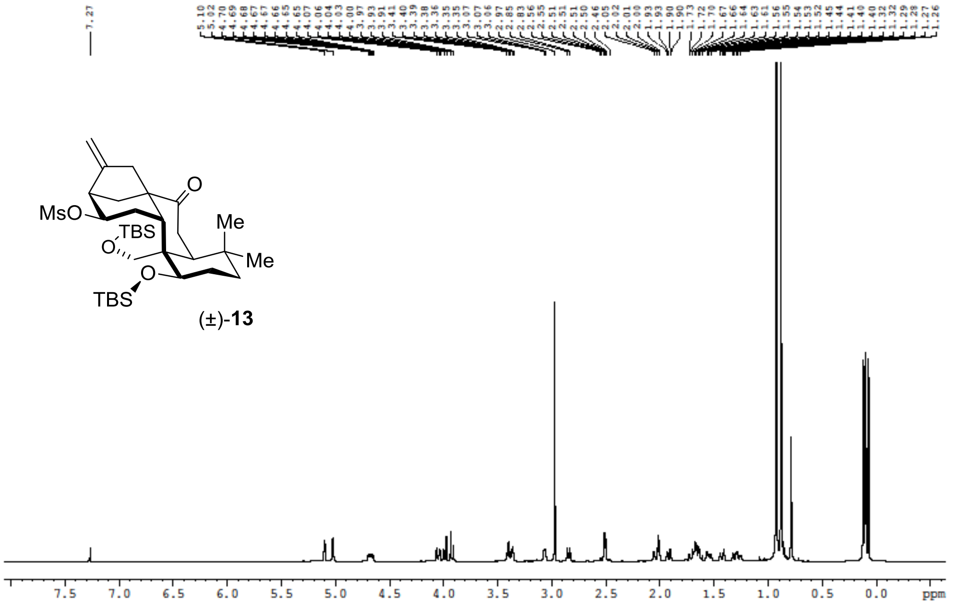

Supplementary Figure 28. ^{1}H NMR of (±)-**13** (400 MHz, $CDCl_3$)

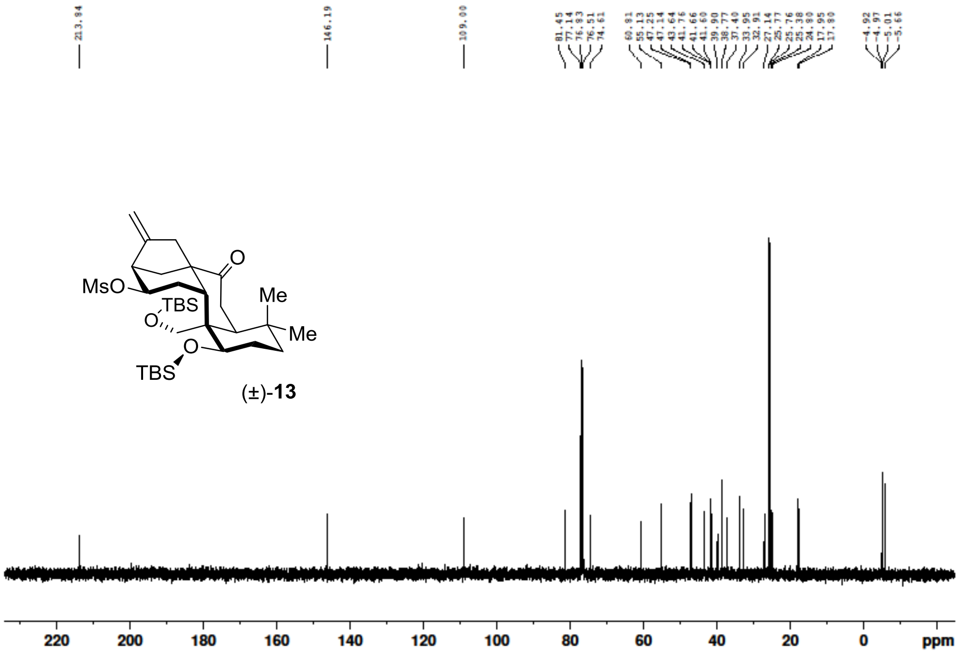

Supplementary Figure 29. ^{13}C NMR of (±)-**12** (101 MHz, $CDCl_3$)

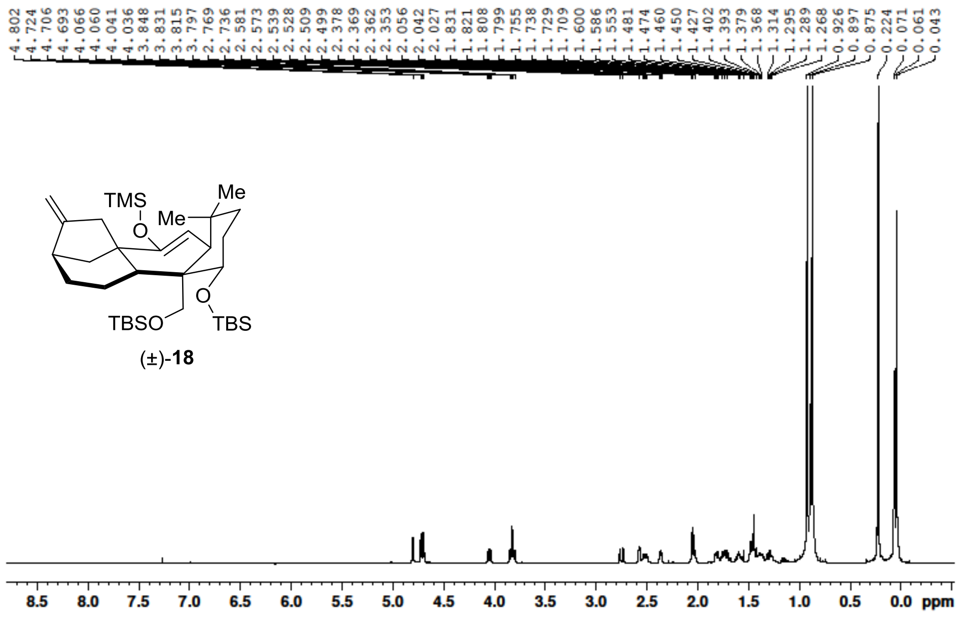

Supplementary Figure 30. ^{1}H NMR of (±)-**18** (500 MHz, $CDCl_3$)

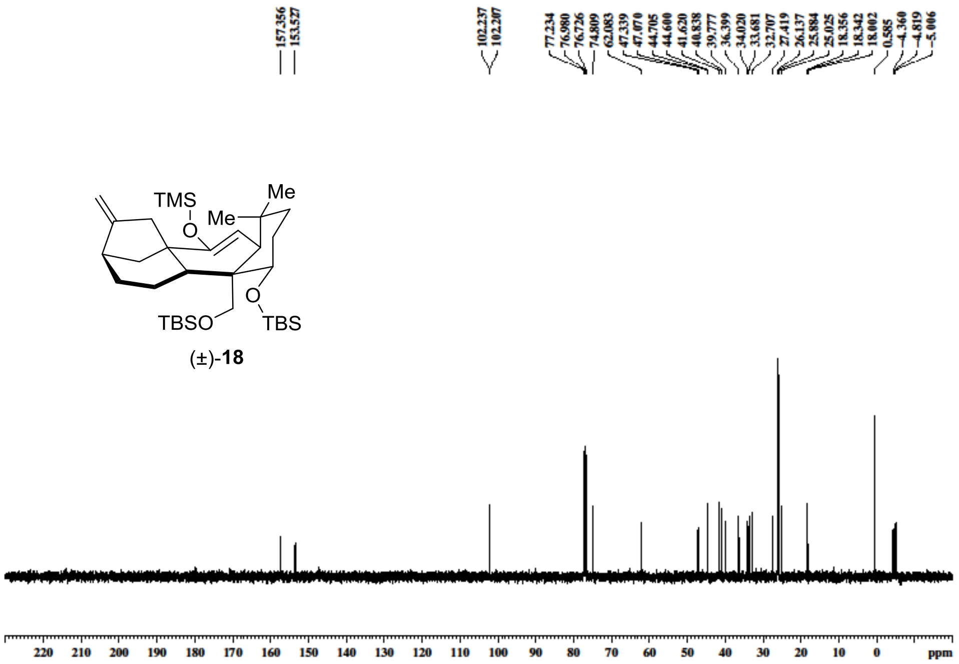

\

Supplementary Figure 31. ^{13}C NMR of (±)-**18** (126 MHz, $CDCl_3$)

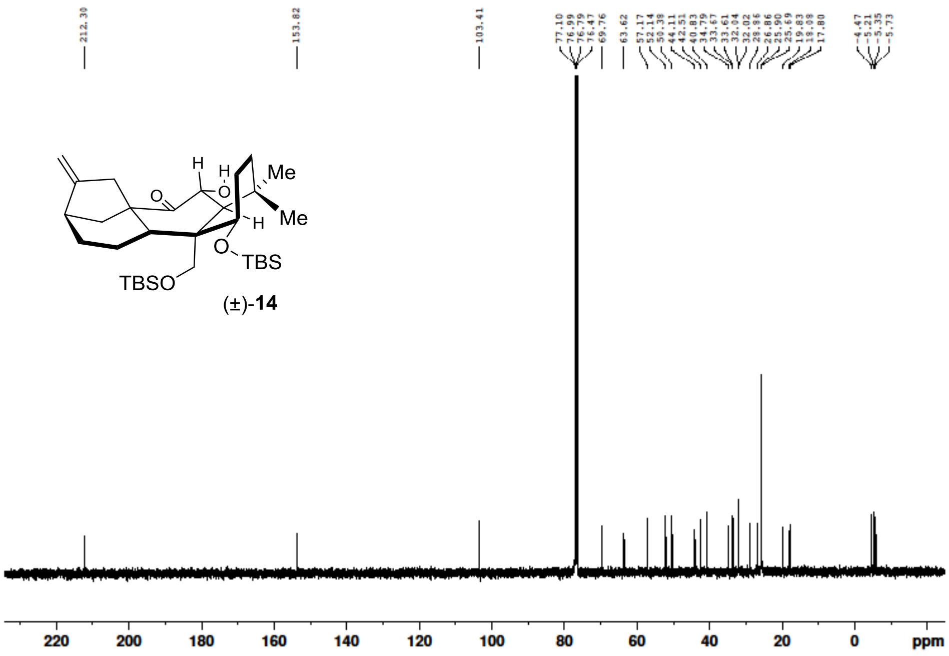

Supplementary Figure 32. ^{1}H NMR of (±)-**14** (400 MHz, $CDCl_3$)

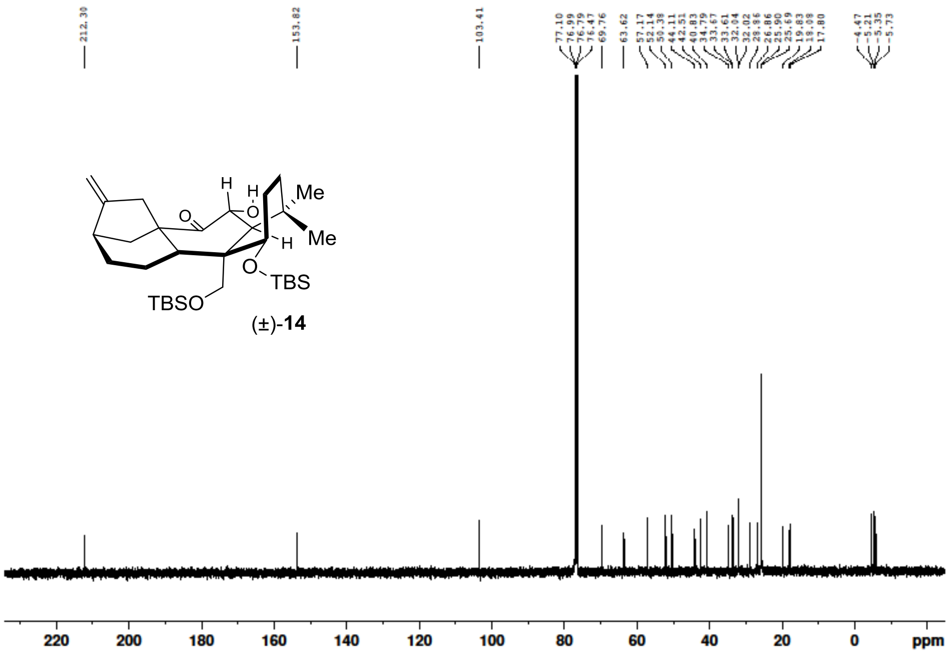

Supplementary Figure 33. ^{13}C NMR of (±)-**14** (101 MHz, $CDCl_3$)

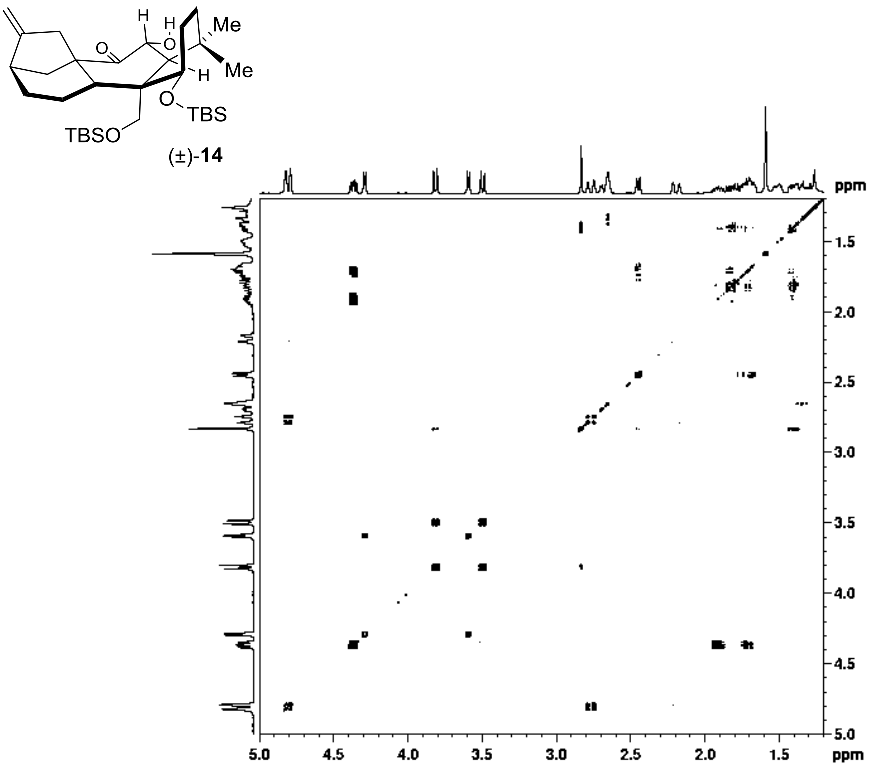

Supplementary Figure 34. COSY NMR of (±)-**14** (400 MHz, $CDCl_3$)

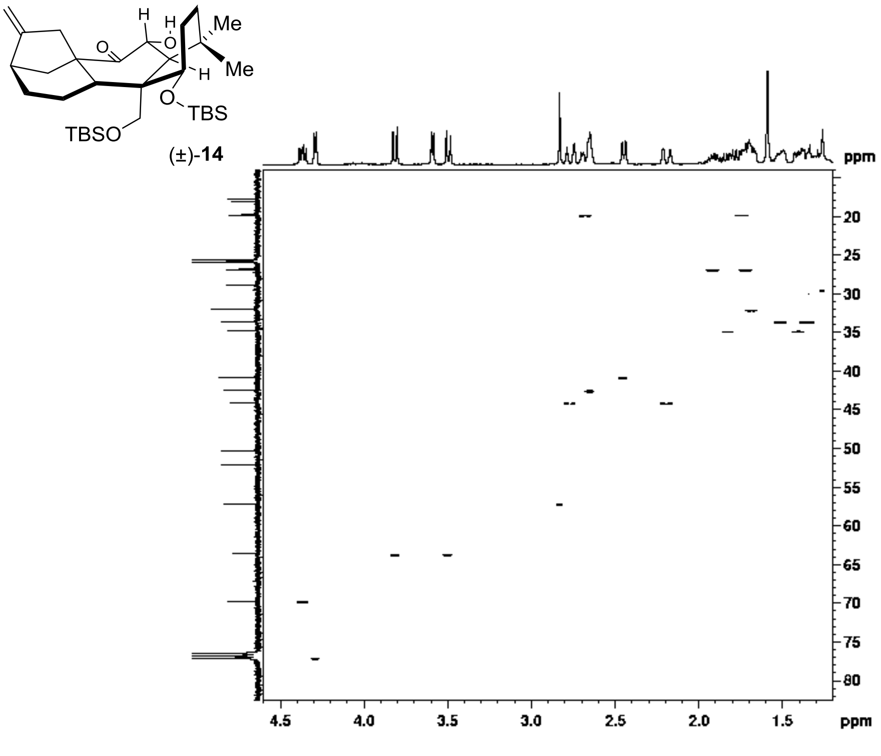

Supplementary Figure 35. HSQC NMR of (±)-**14** (400 MHz, $CDCl_3$)

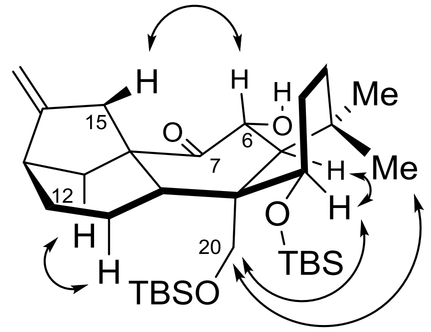

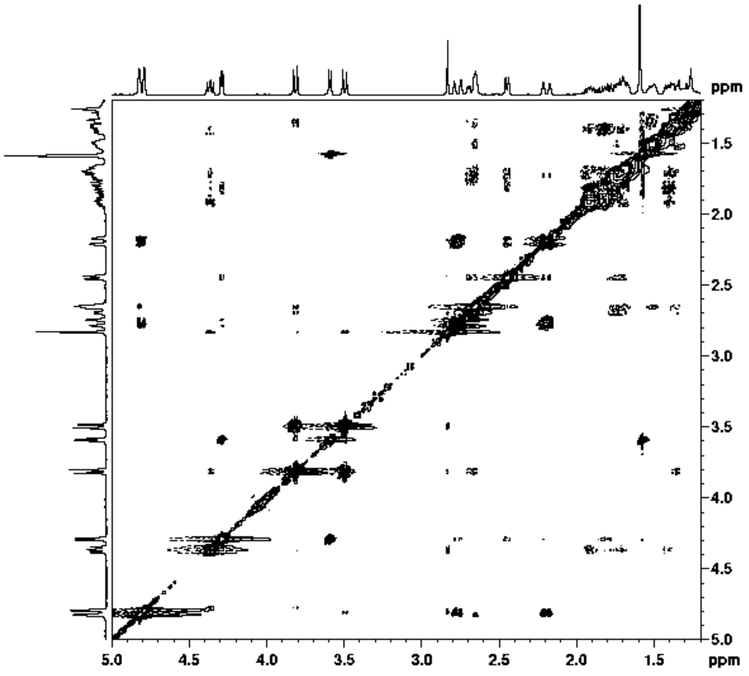

Supplementary Figure 36. NOSEY NMR of (±)-**14** (400 MHz, $CDCl_3$)

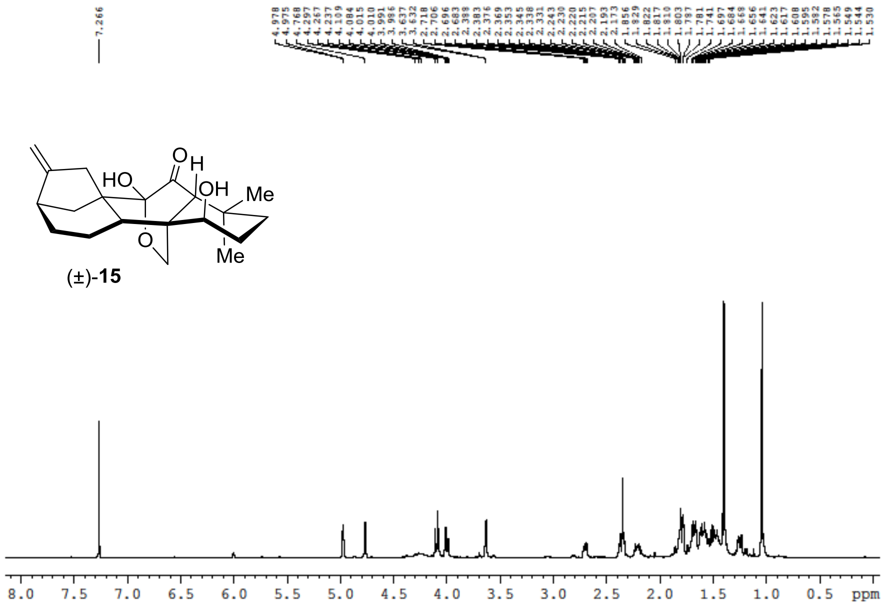

Supplementary Figure 37. ^{1}H NMR of (±)-**15** (400 MHz, $CDCl_3$)

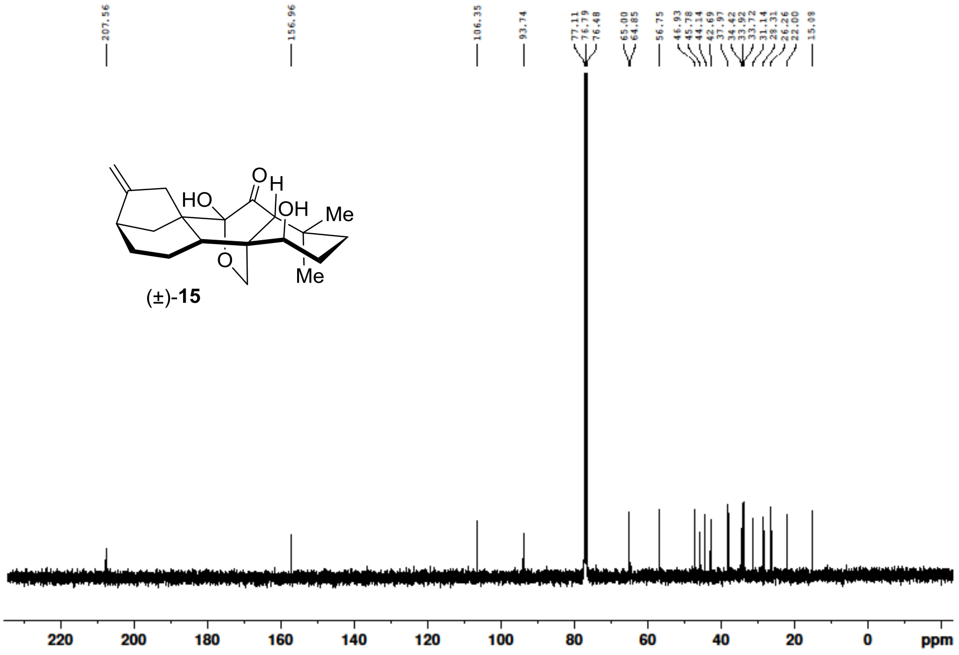

Supplementary Figure 38. ^{13}C NMR of (±)-**15** (101 MHz, $CDCl_3$)

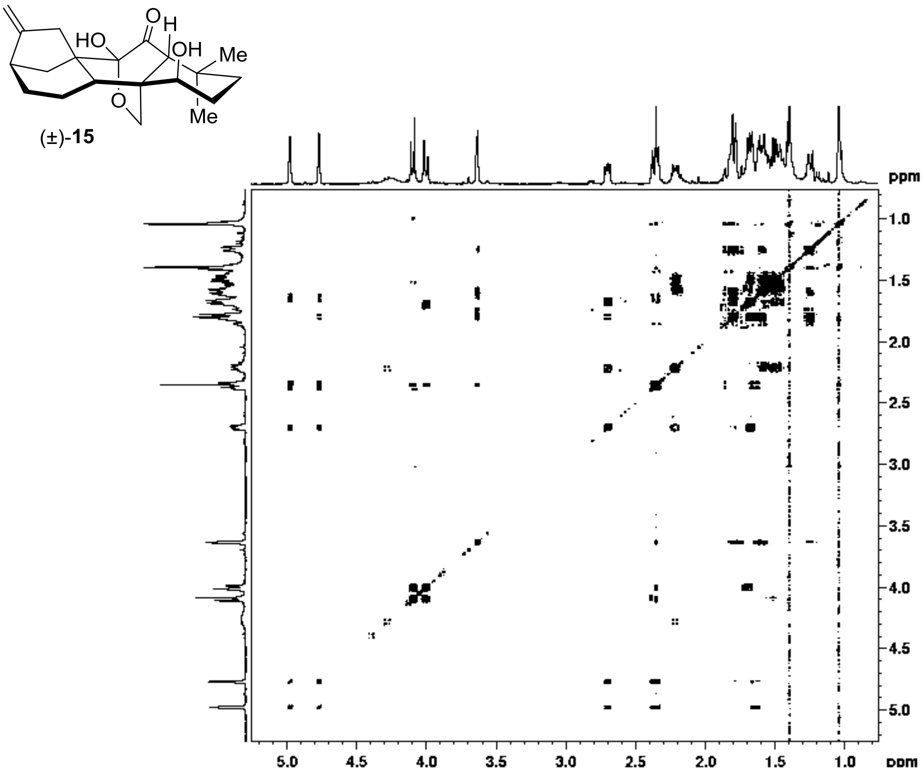

Supplementary Figure 39. COSY NMR of (±)-**15** (400 MHz, $CDCl_3$)

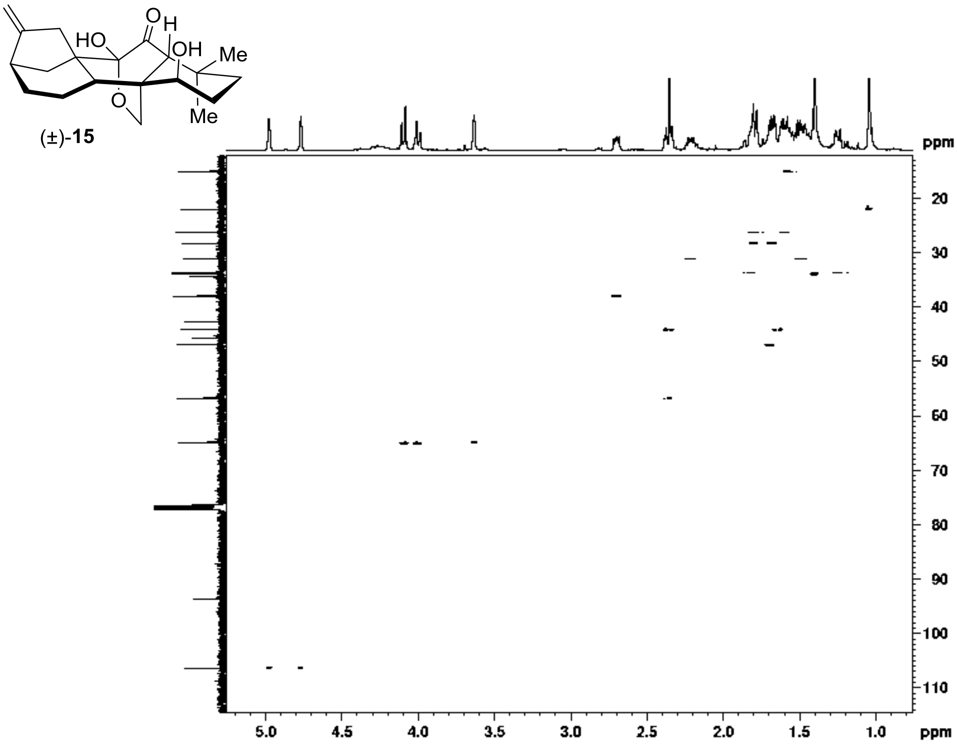

Supplementary Figure 40. HSQC NMR of (±)-**15** (400 MHz, $CDCl_3$)

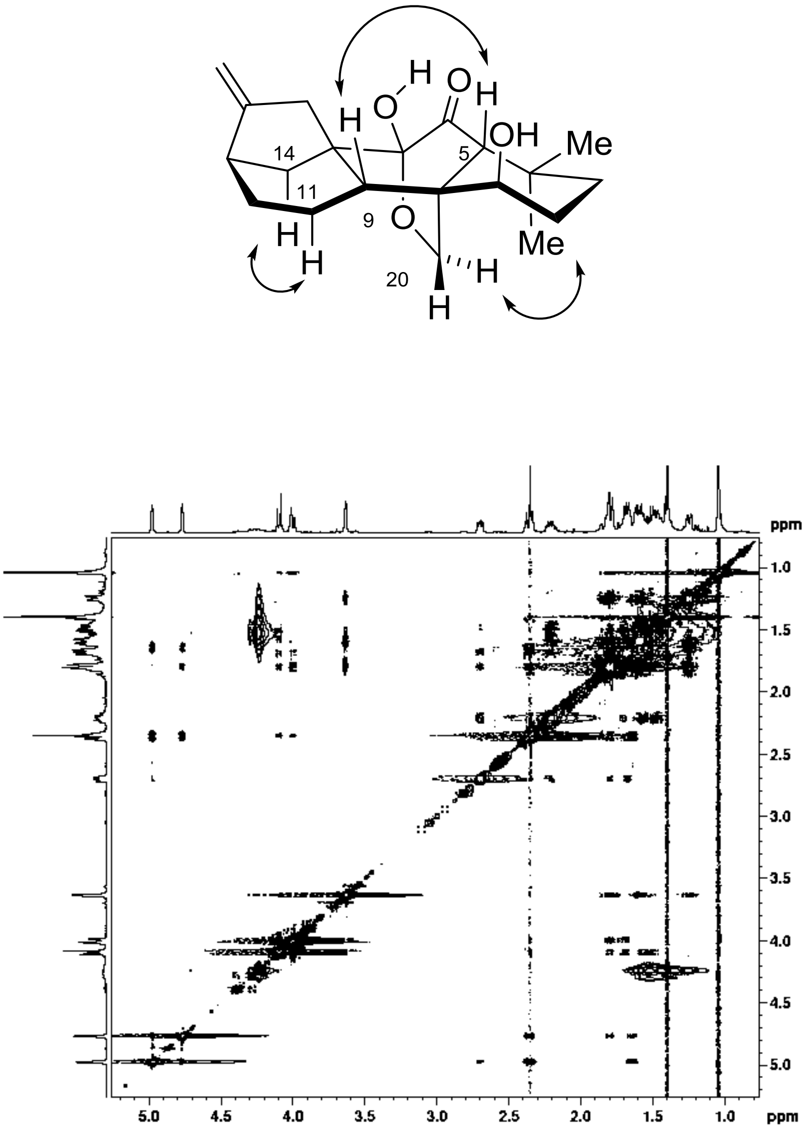

Supplementary Figure 41. NOSEY NMR of (±)-**15** (400 MHz, $CDCl_3$)

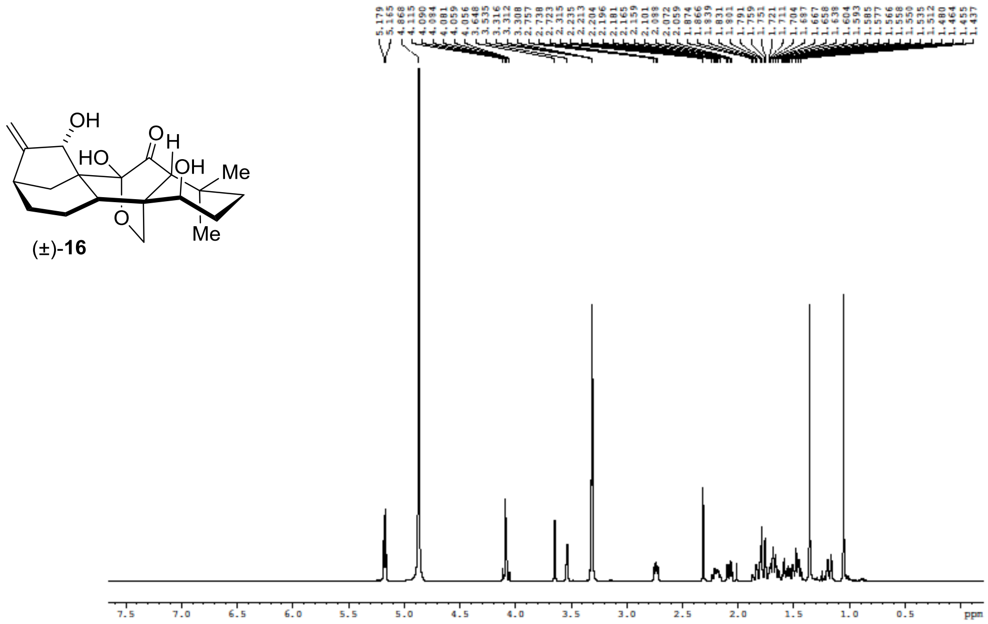

Supplementary Figure 42. ^{1}H NMR of (±)-**16** (500 MHz, CD_3OD)

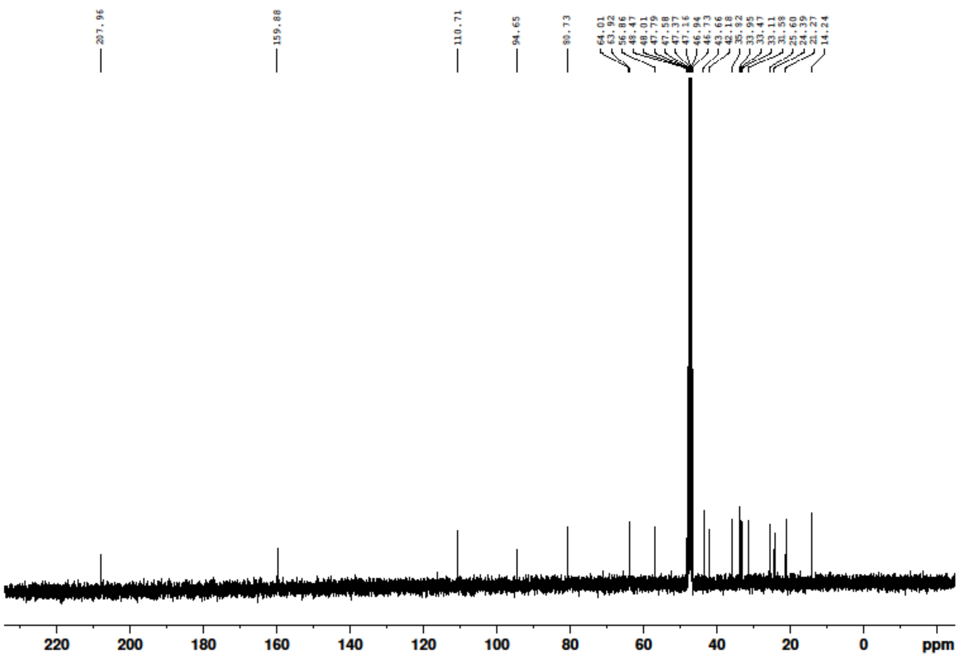

Supplementary Figure 43. ^{13}C NMR of (±)-**16** (126 MHz, CD_3OD)

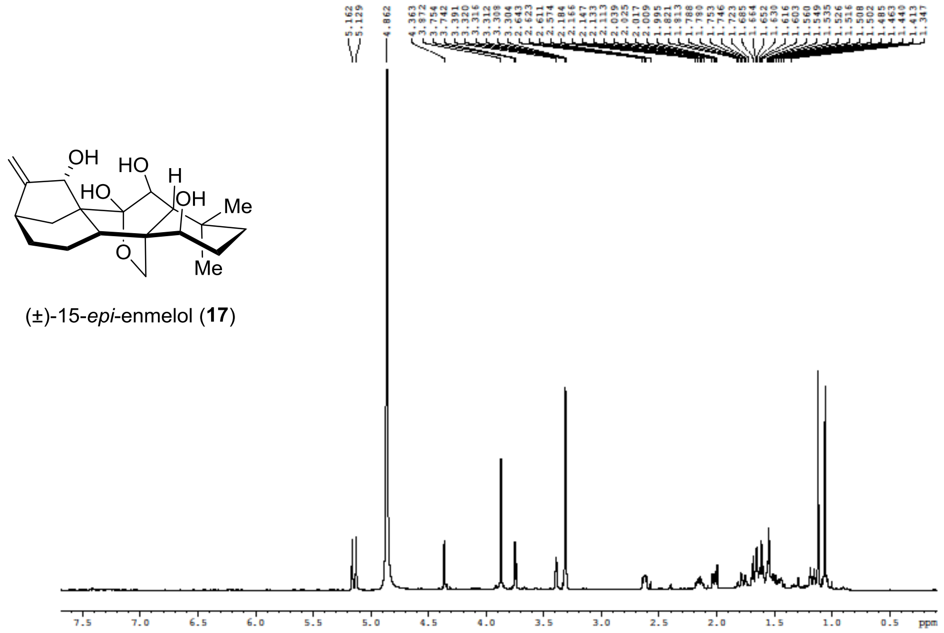

Supplementary Figure 44. ^{1}H NMR of (±)-**17** (500 MHz, CD_3OD)

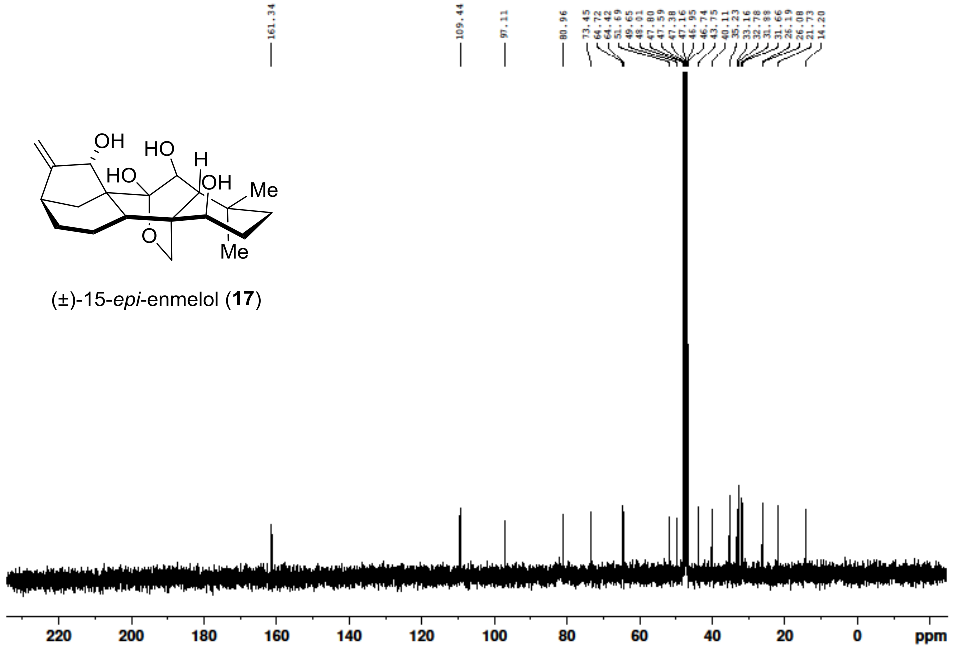

Supplementary Figure 45. ^{13}C NMR of (±)-**17** (126 MHz, CD_3OD)

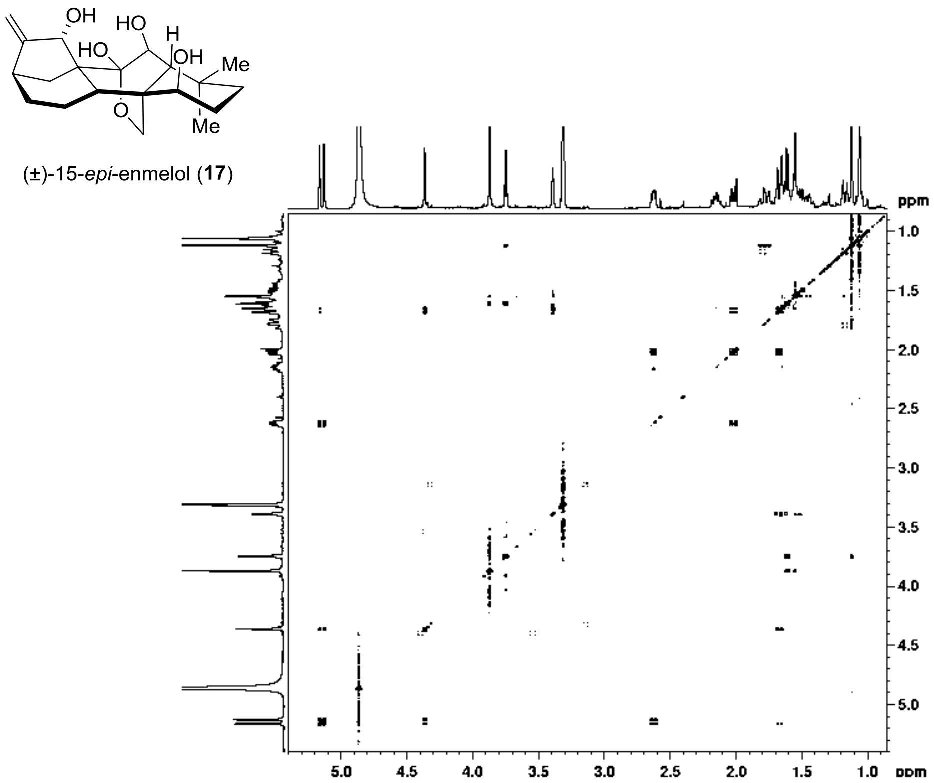

Supplementary Figure 46. COSY NMR of (±)-**17** (500 MHz, CD_3OD)

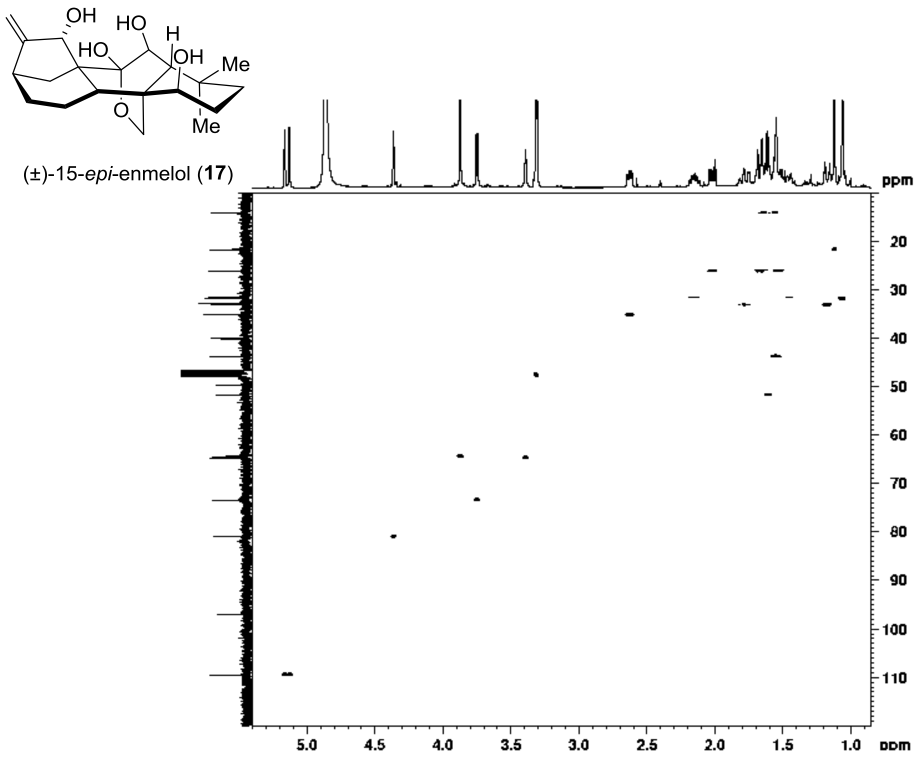

Supplementary Figure 47. HSQC NMR of (±)-**17** (500 MHz, CD_3OD)

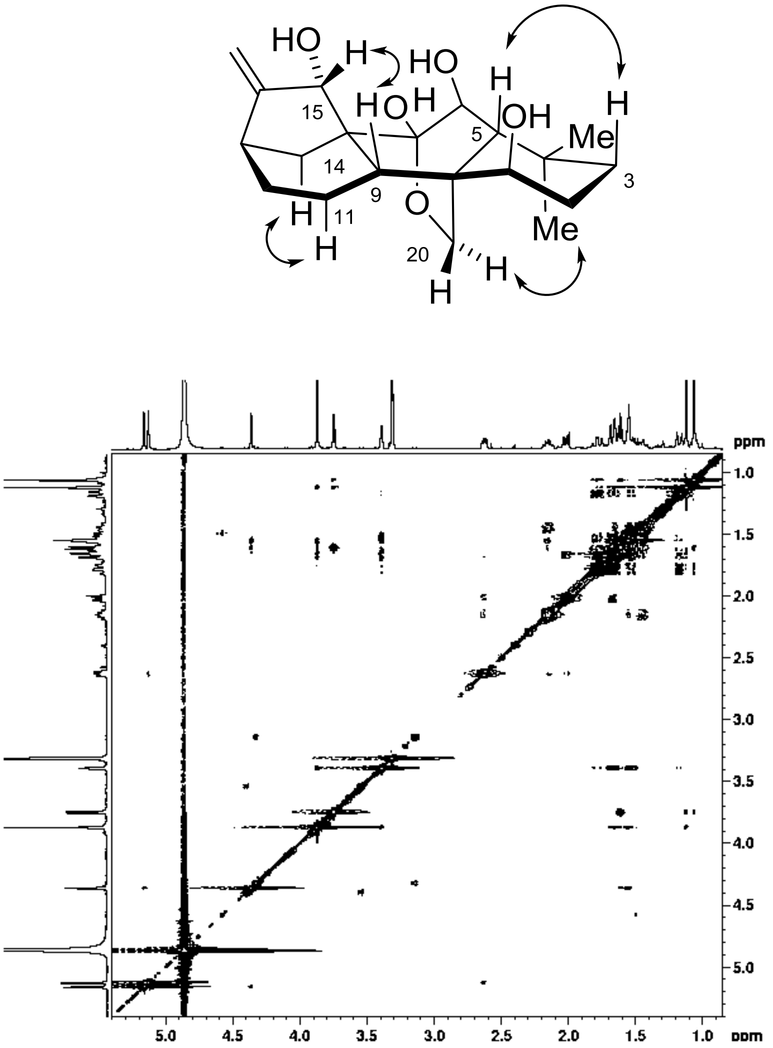

Supplementary Figure 48. NOSEY NMR of (±)-**17** (500 MHz, CD_3OD)

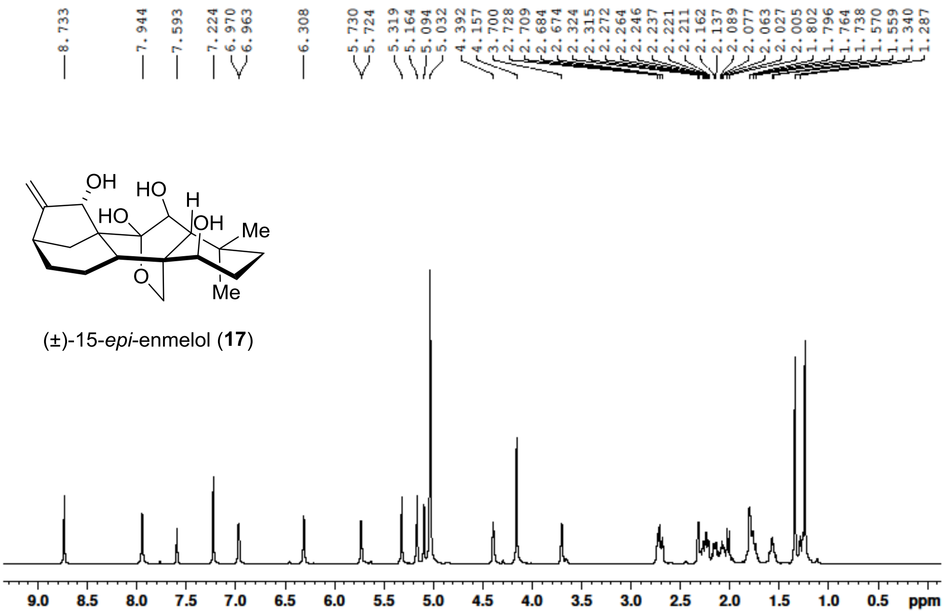

Supplementary Figure 49. ^{1}H NMR of (±)-**17** (500 MHz, pyridine-d_5)

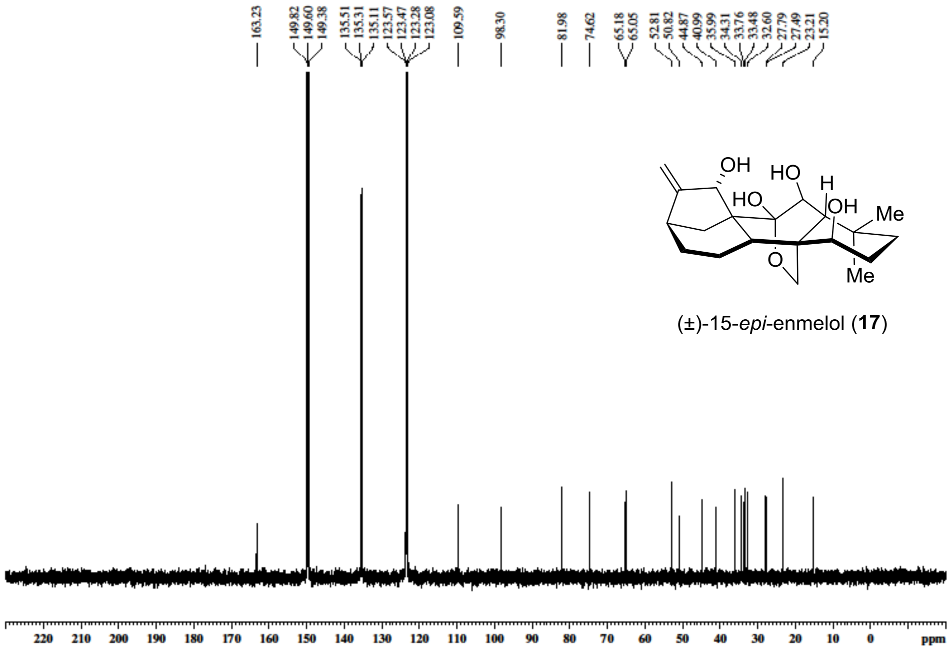

Supplementary Figure 50. ^{13}C NMR of (±)-**17** (126 MHz, pyridine-d_5)

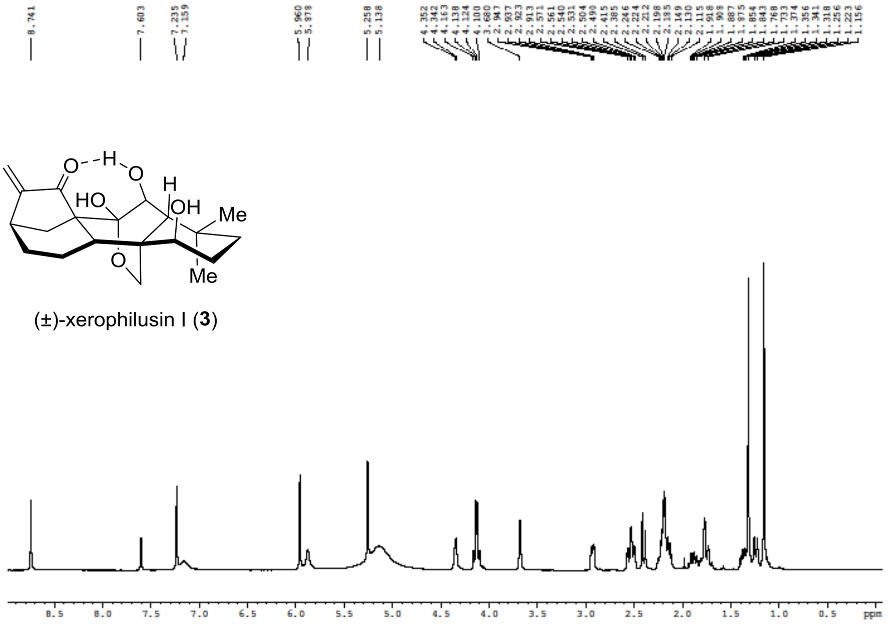

Supplementary Figure 51. ^{1}H NMR of (±)-**3** (400 MHz, pyrdieine-d_5)

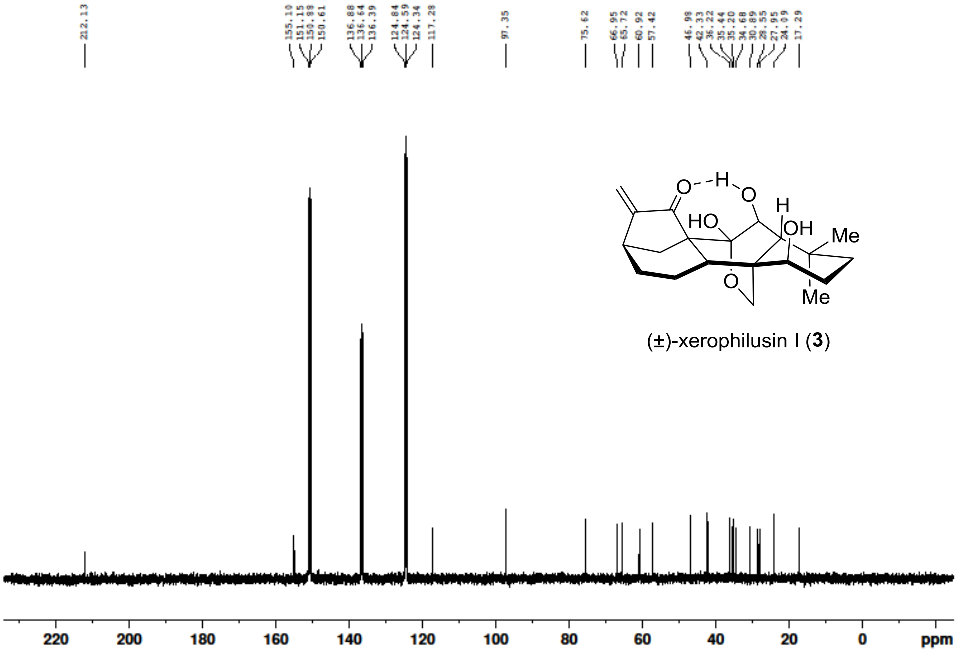

Supplementary Figure 52. ^{13}C NMR of (±)-**3** (101 MHz, pyrdieine-d_5)

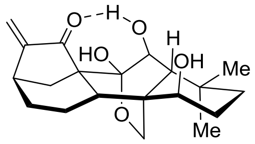

(±)-xerophilusin I (**3**)

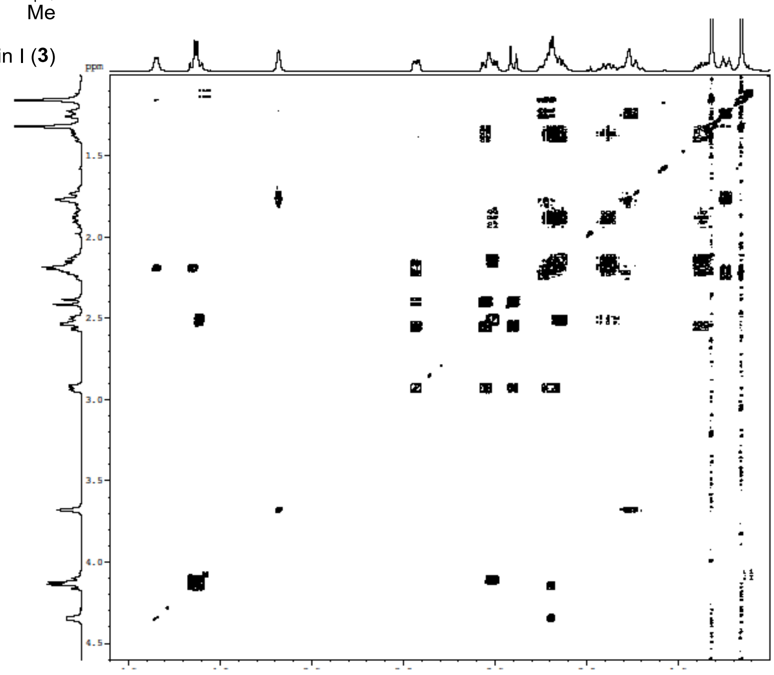

Supplementary Figure 53. COSY NMR of (±)-**3** (400 MHz, pyrdieine-d_5)

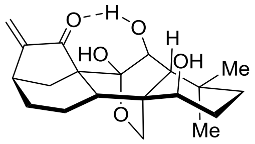

(±)-xerophilusin I (**3**)

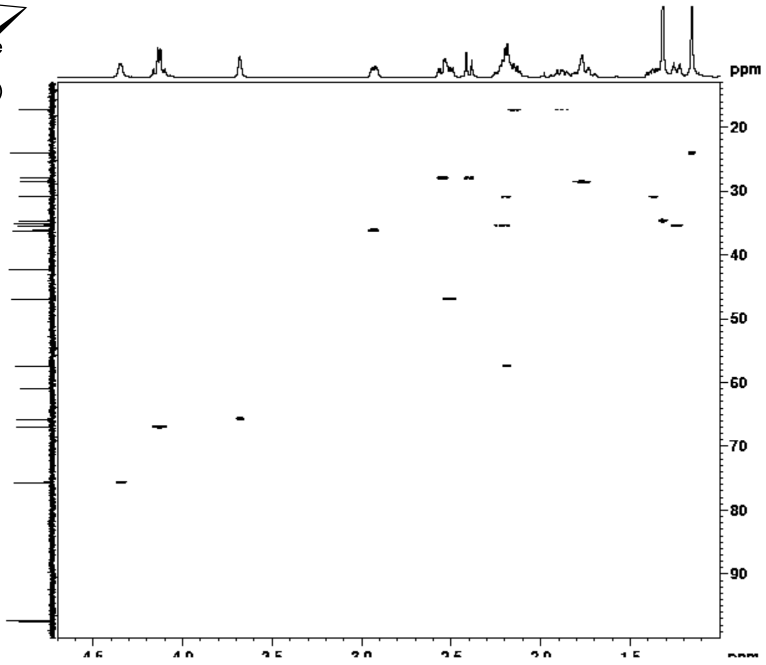

Supplementary Figure 54. HSQC NMR of (±)-**3** (400 MHz, pyrdieine-d_5)

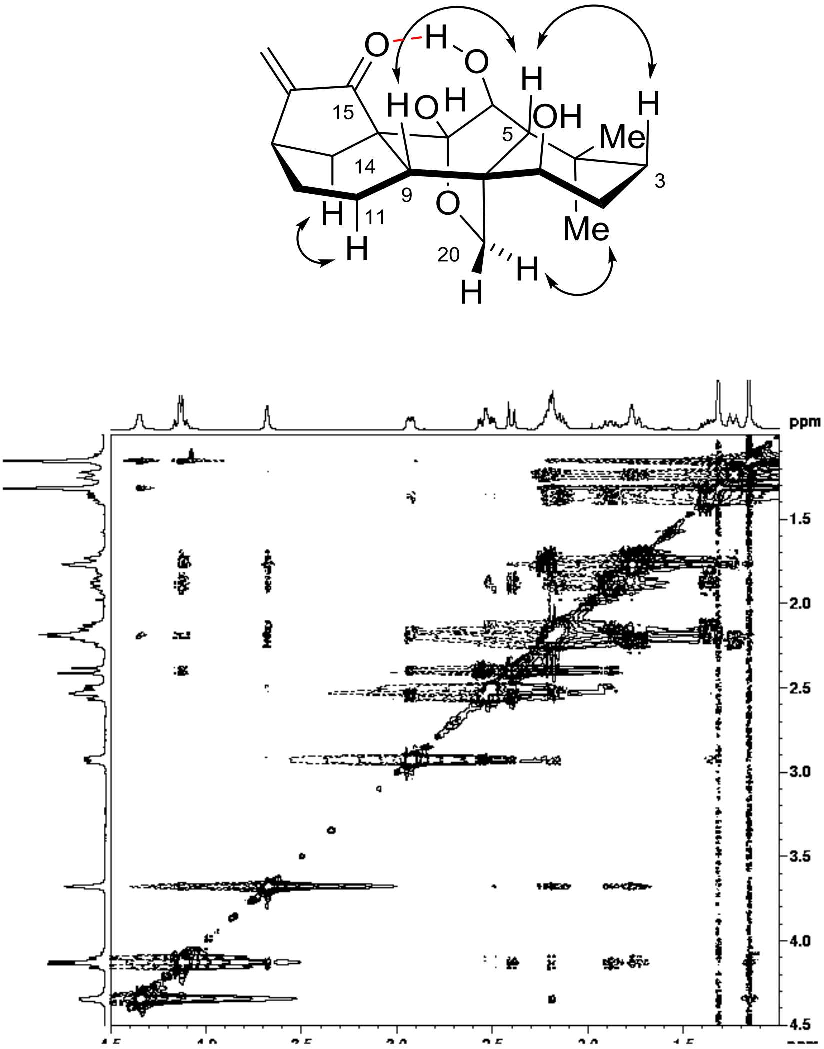

Supplementary Figure 55. NOSEY NMR of (±)-**3** (400 MHz, pyrdieine-d_5)

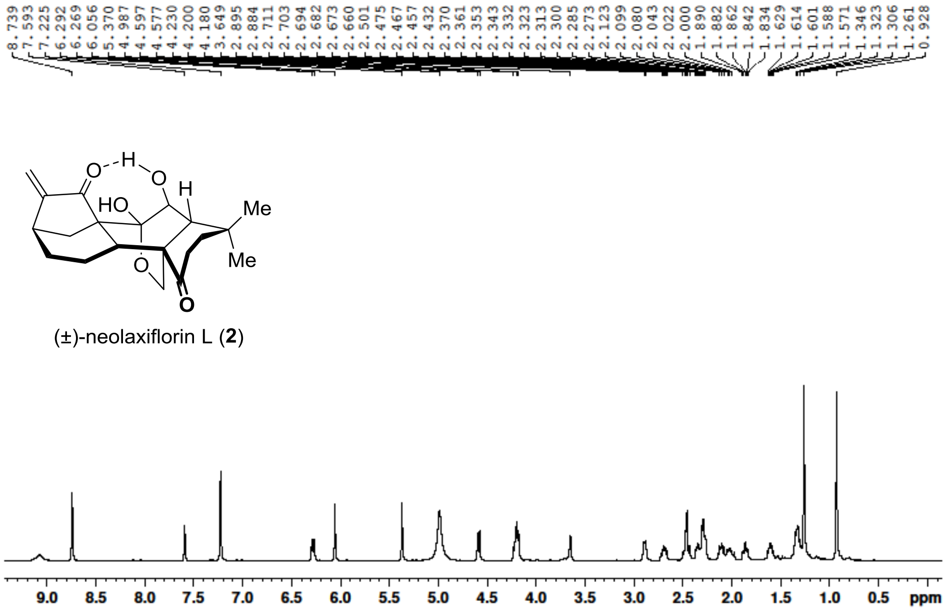

Supplementary Figure 56. ^{1}H NMR of (±)-**2** (500 MHz, pyrdieine-d_5)

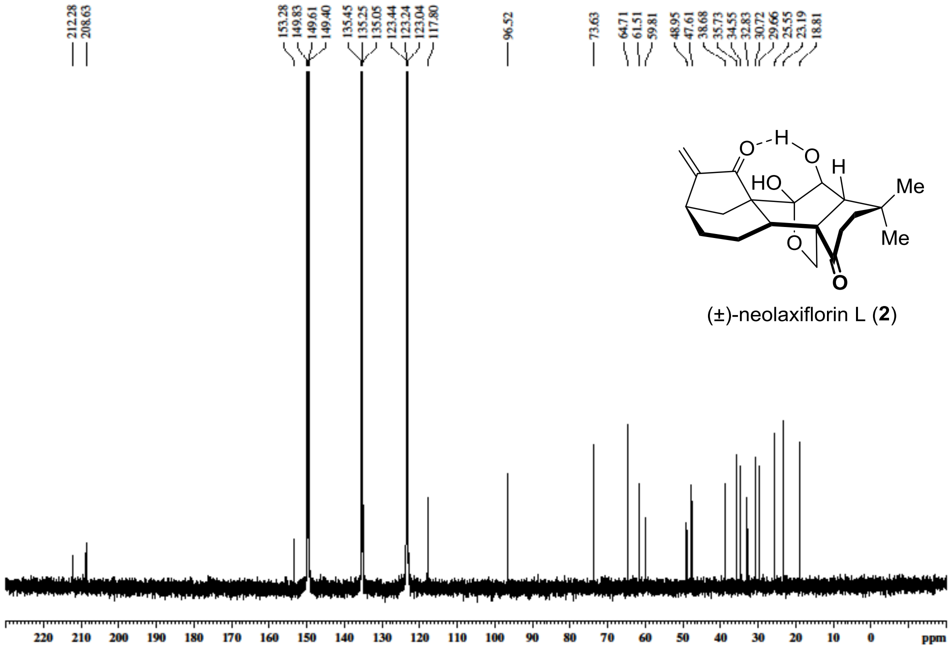

Supplementary Figure 57. ^{13}C NMR of (±)-**2** (126 MHz, pyrdieine-d_5)

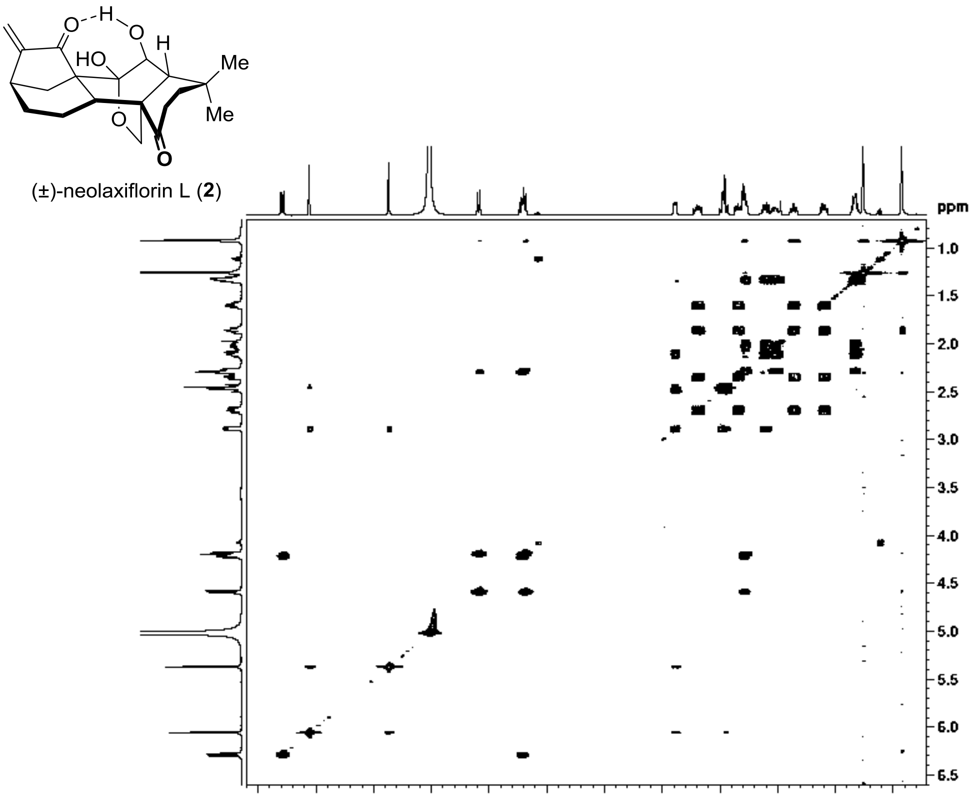

Supplementary Figure 58. COSY NMR of (±)-**2** (500 MHz, pyrdieine-d_5)

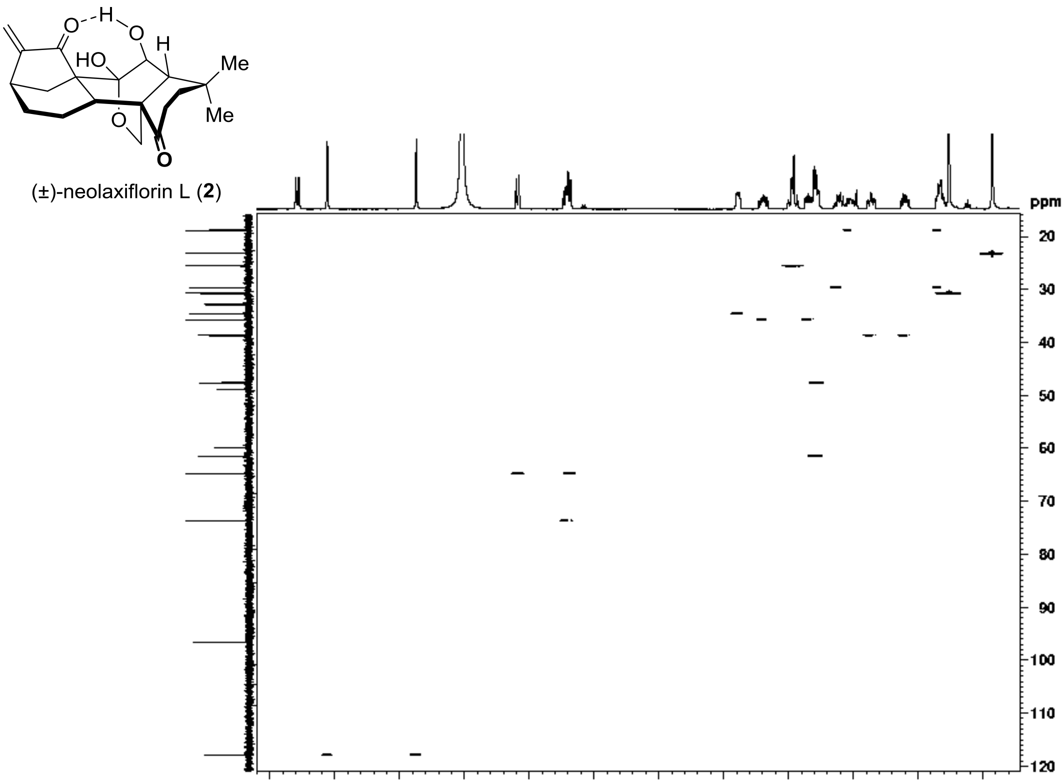

Supplementary Figure 59. HSQC NMR of (±)-**2** (500 MHz, pyrdieine-d_5)

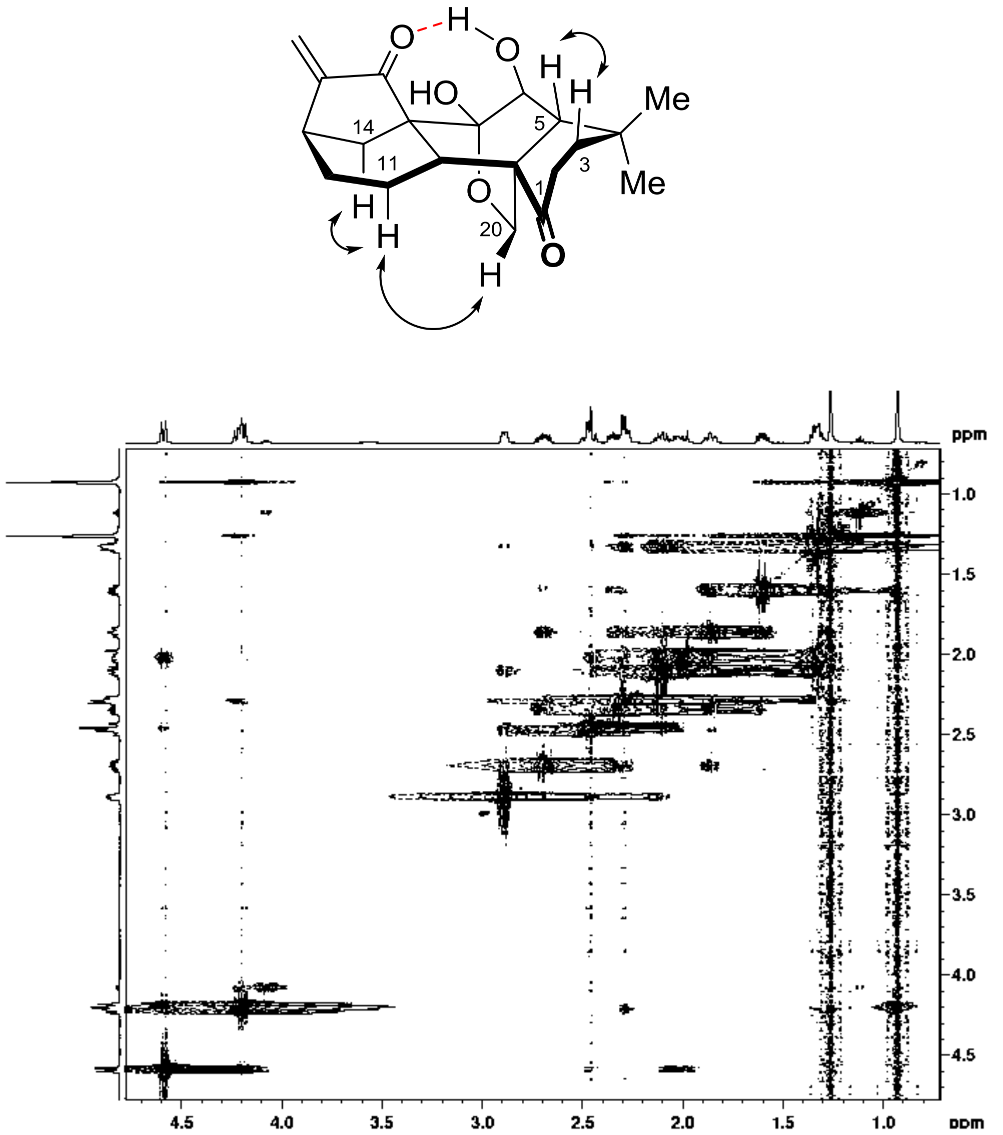

Supplementary Figure 60. NOSEY NMR of (±)-**2** (500 MHz, pyrdieine-d_5)

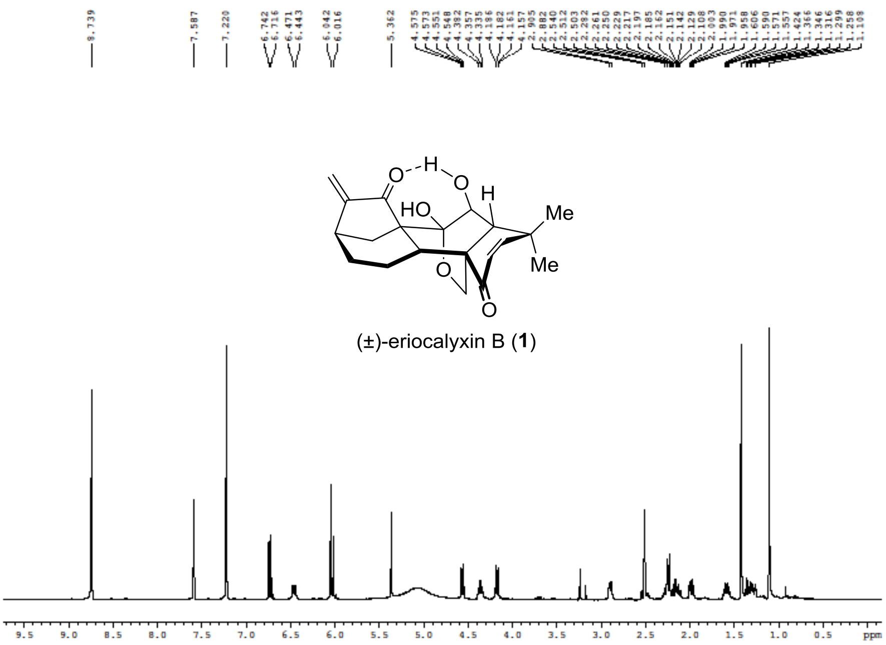

Supplementary Figure 61. ^{1}H NMR of (±)-**1** (400 MHz, $CDCl_3$)

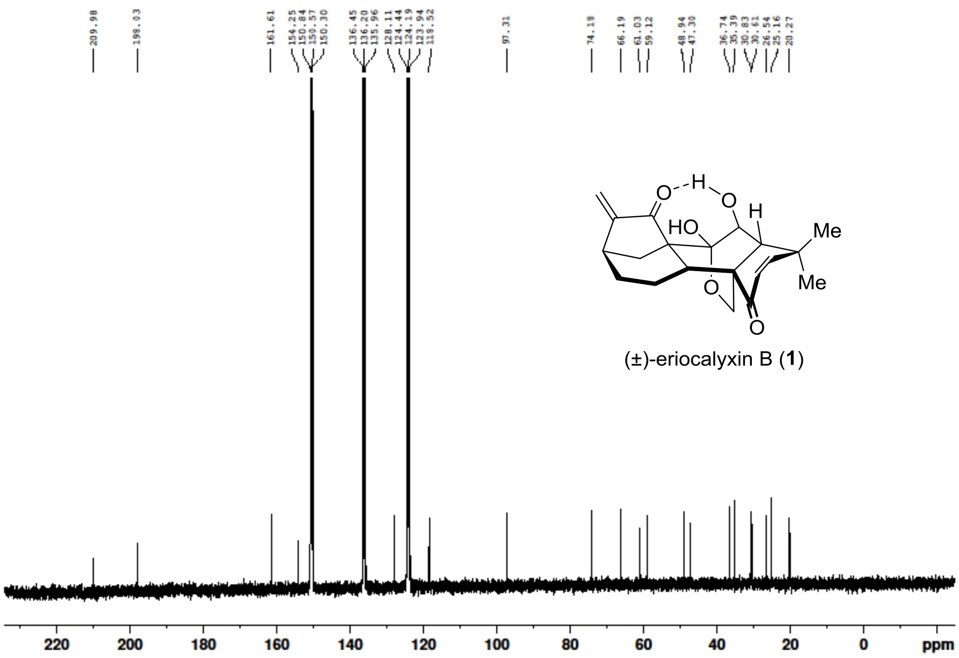

Supplementary Figure 62. ^{13}C NMR of (±)-**1** (101 MHz, $CDCl_3$)

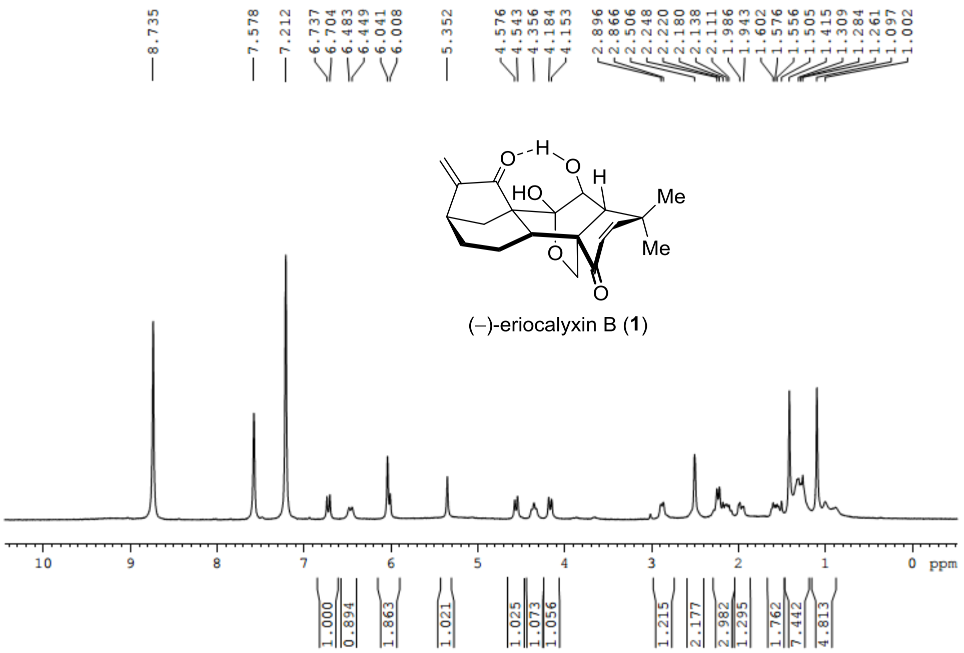

Supplementary Figure 63. ^{1}H NMR of (–)-**1** (300 MHz, pyridine-d_5)

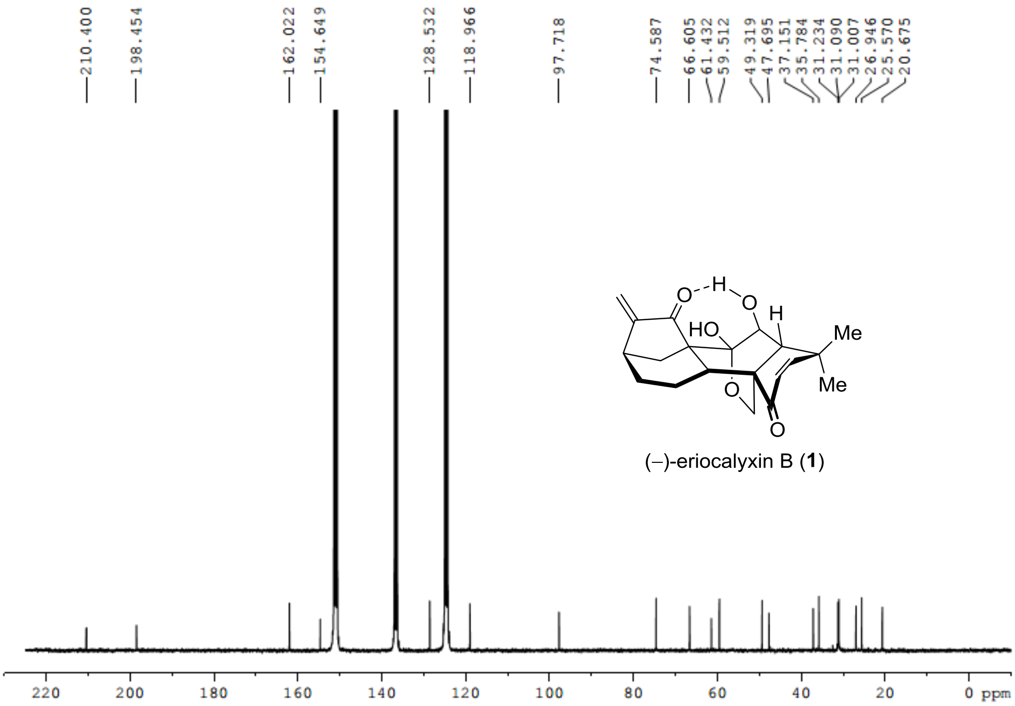

Supplementary Figure 64. ^{13}C NMR of (–)-**1** (76 MHz, pyridine-d_5)

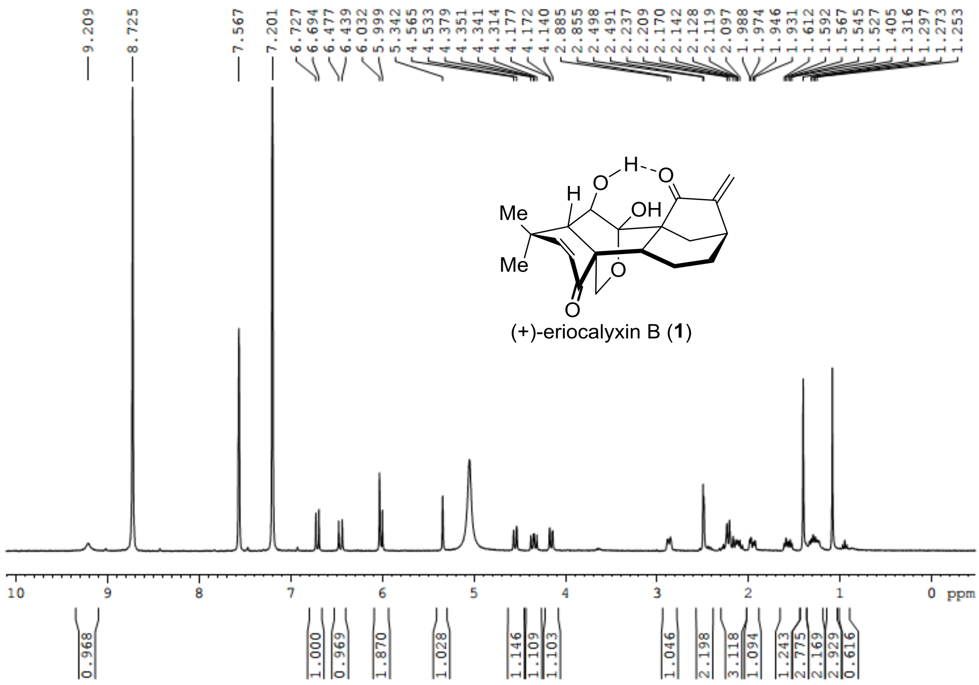

Supplementary Figure 65. ^{1}H NMR of (+)-**1** (300 MHz, pyridine-d_5)

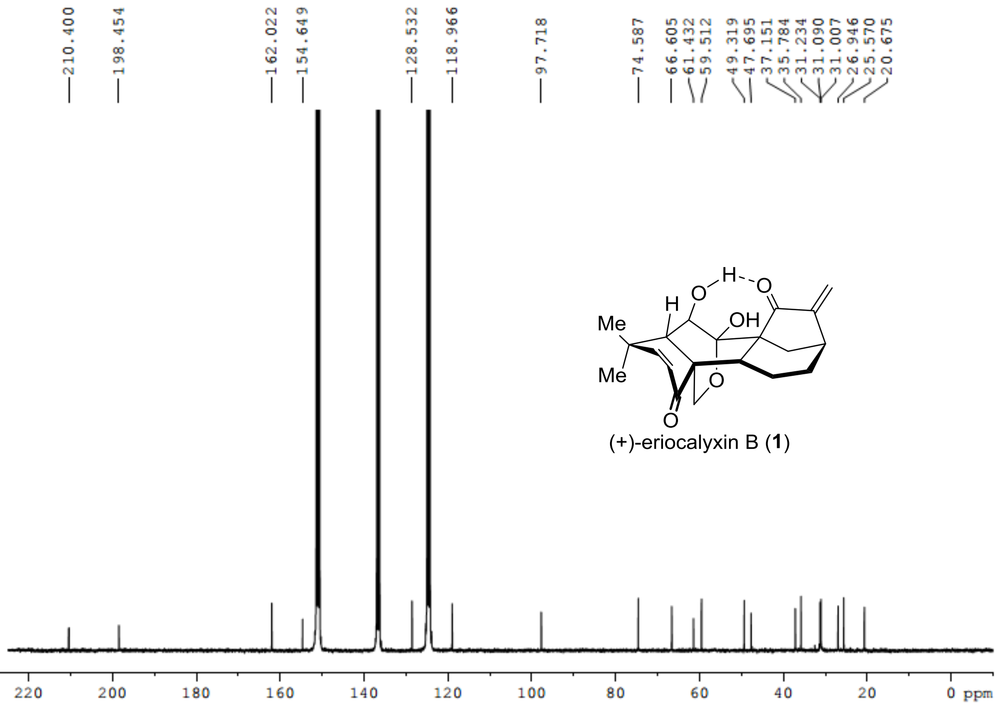

Supplementary Figure 66. ^{13}C NMR of (+)-**1** (126 MHz, pyridine-d_5)

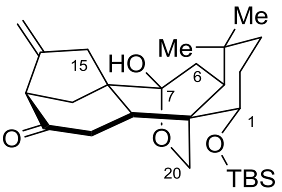

(±)-**12**

This compound was recrystallized from hexanes and ethyl acetate.

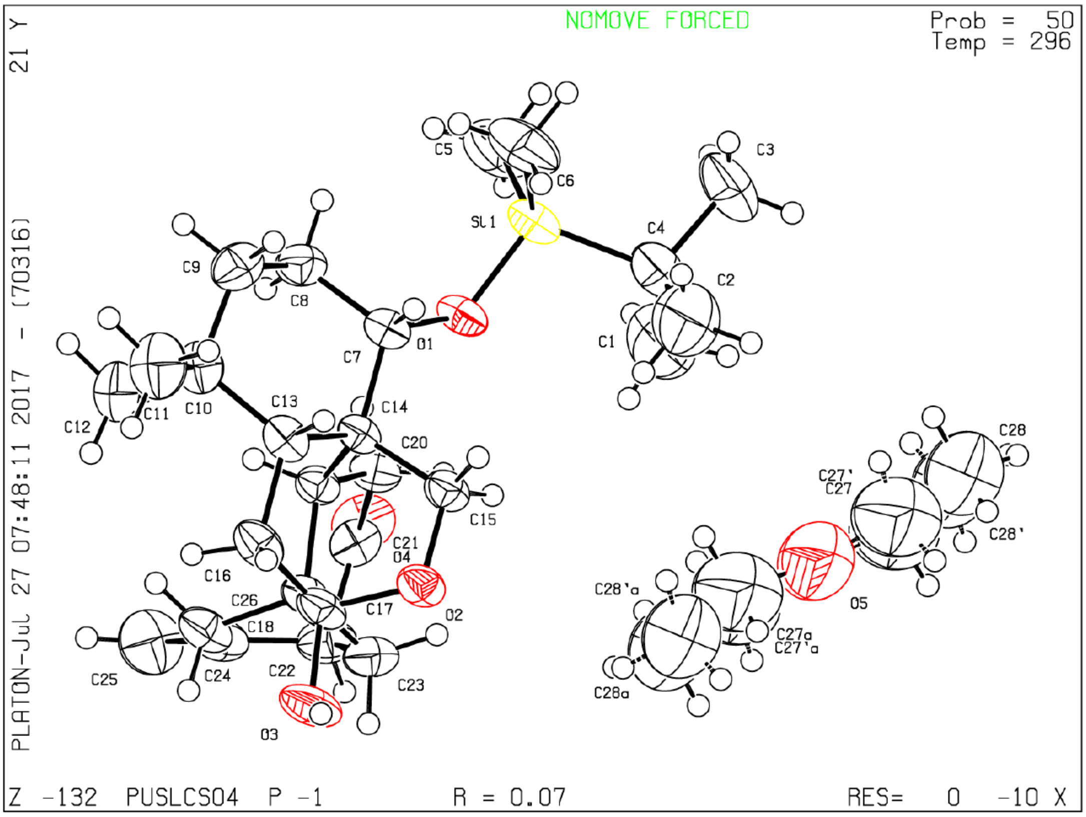

Supplementary Figure 67. ORTEP presentation of (±)-**12**

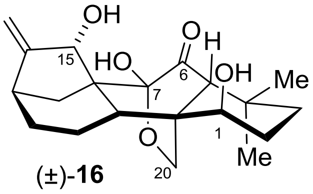

(±)-**16**

This compound was recrystallized from THF, ethyl acetate and hexanes.

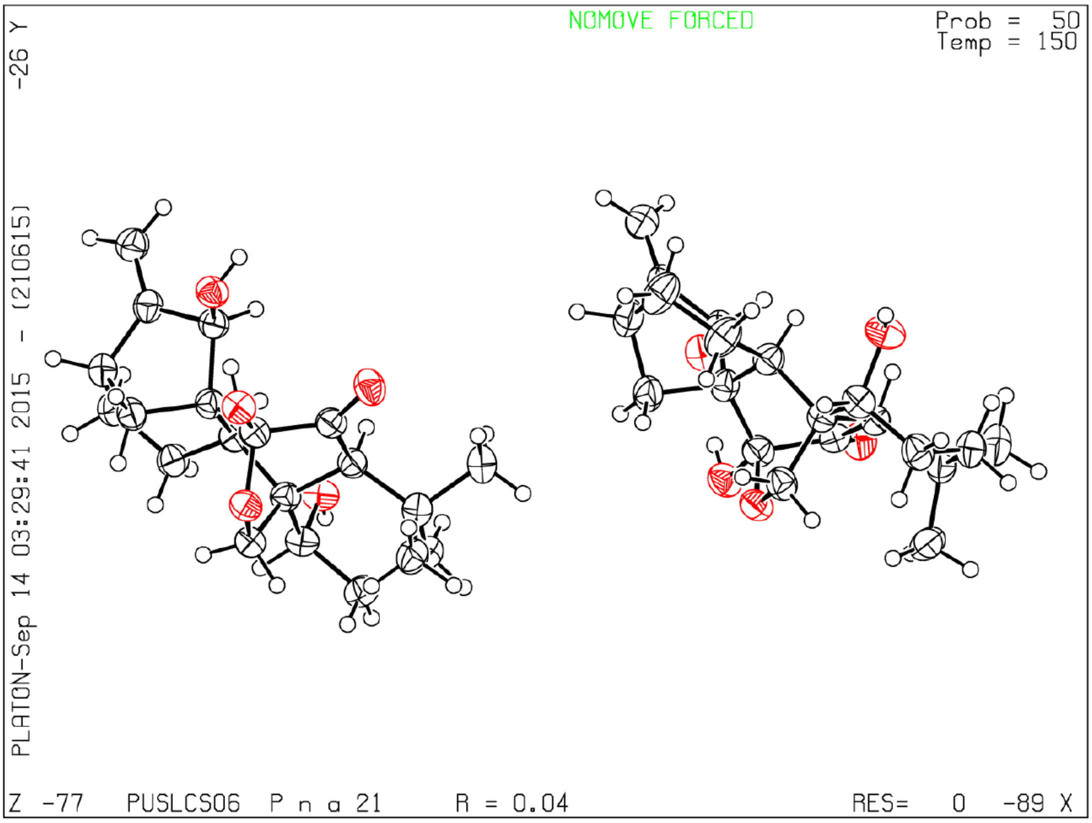

Supplementary Figure 68. ORTEP presentation of (±)-**16**

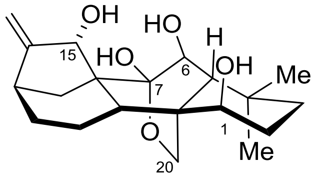

(±)-15-*epi*-enmelol (**17**)

This compound was recrystallized from THF, ethyl acetate and hexanes.

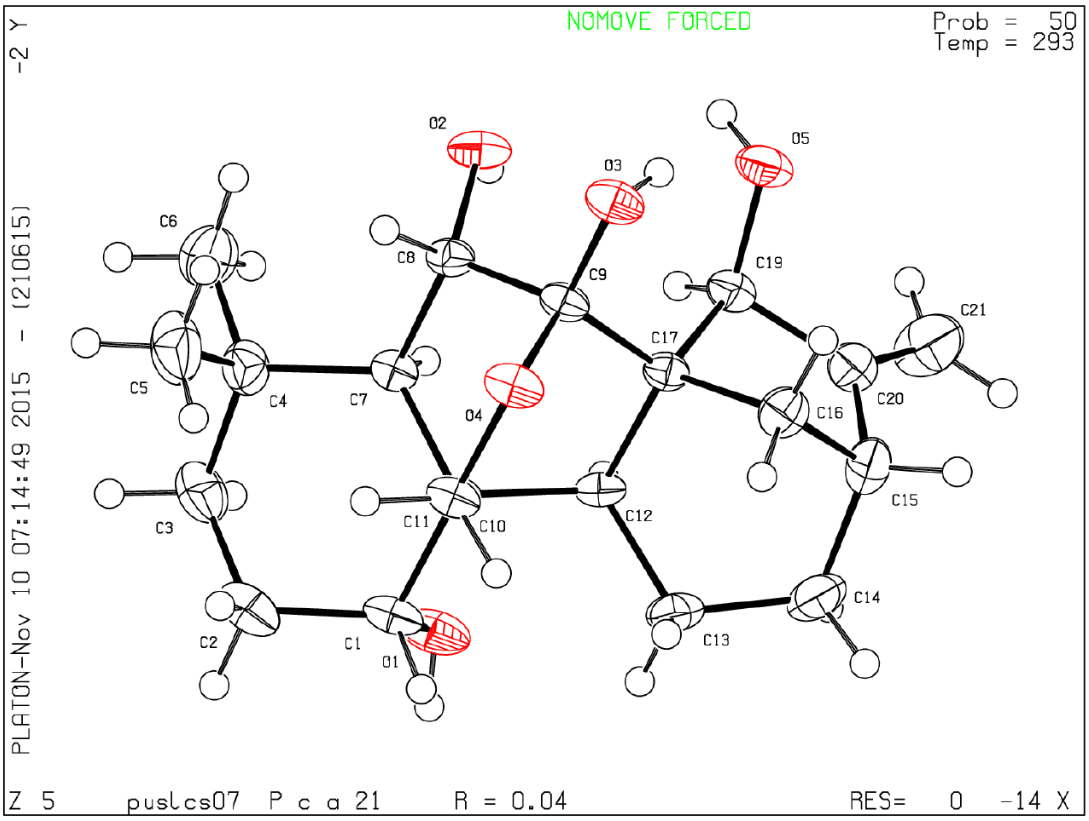

Supplementary Figure 69. ORTEP presentation of (±)-**17**

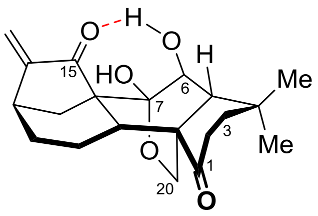

(±)-neolaxiflorin L (**2**)

This compound was recrystallized from THF and hexanes.

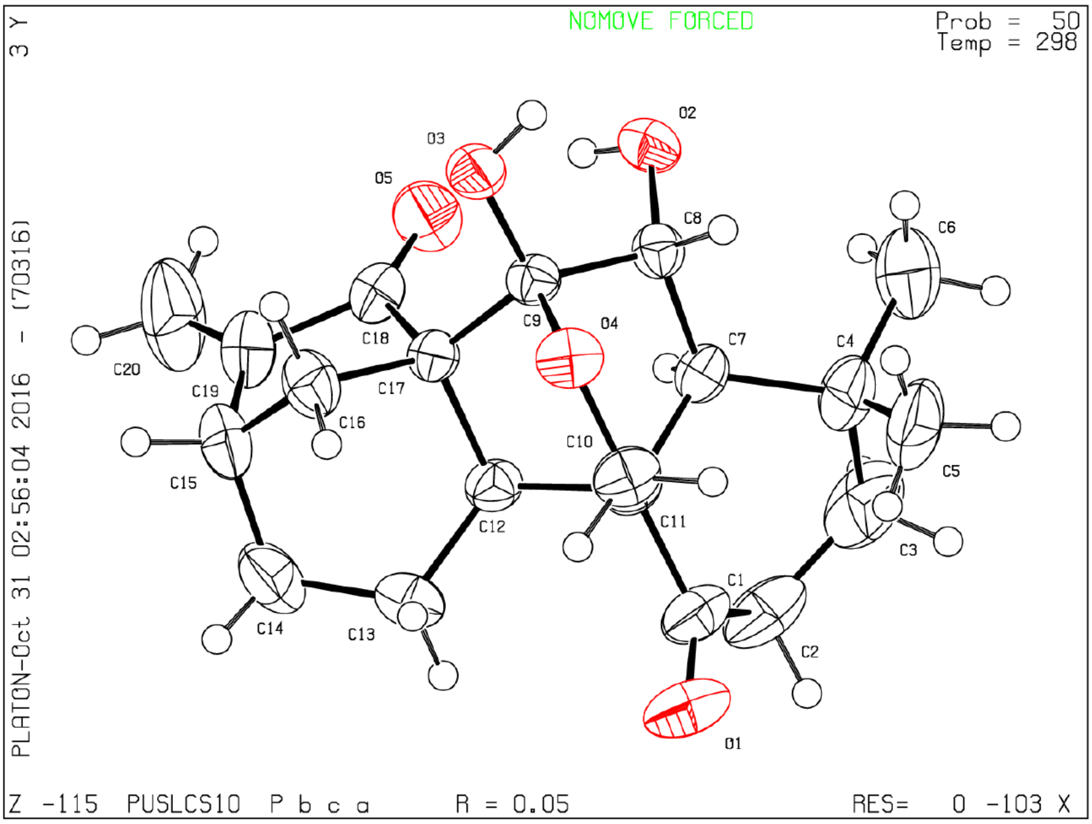

Supplementary Figure 70. ORTEP presentation of (±)-**2**

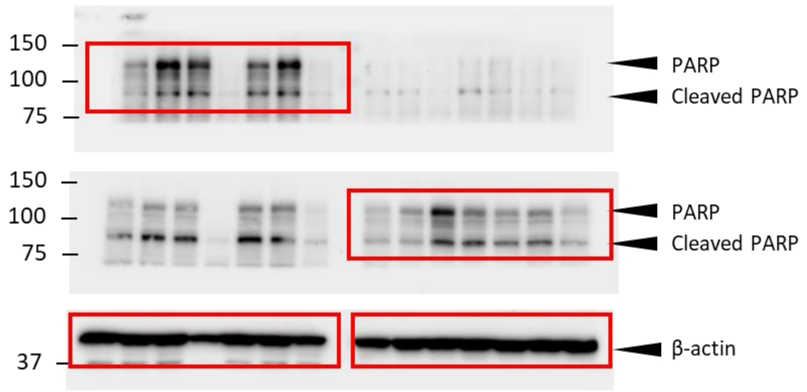

Supplementary Figure 71. Unprocessed and uncropped scans of original blots shown in Supplementary Figure 2A.

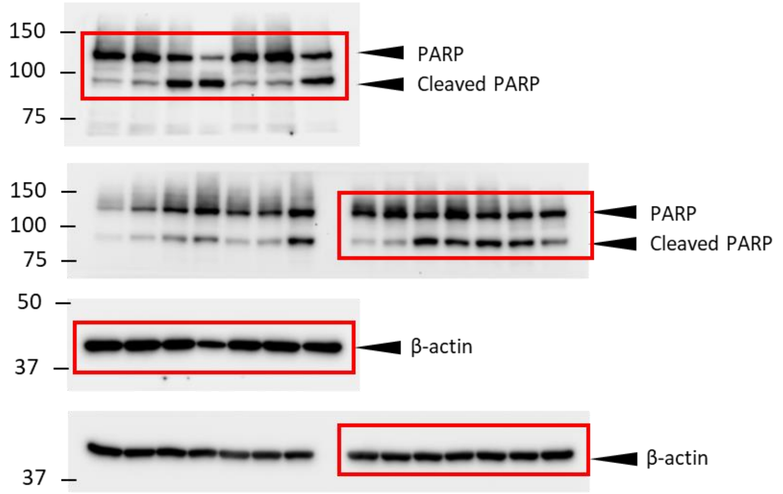

Supplementary Figure 72. Unprocessed and uncropped scans of original blots shown in Supplementary Figure 2B.

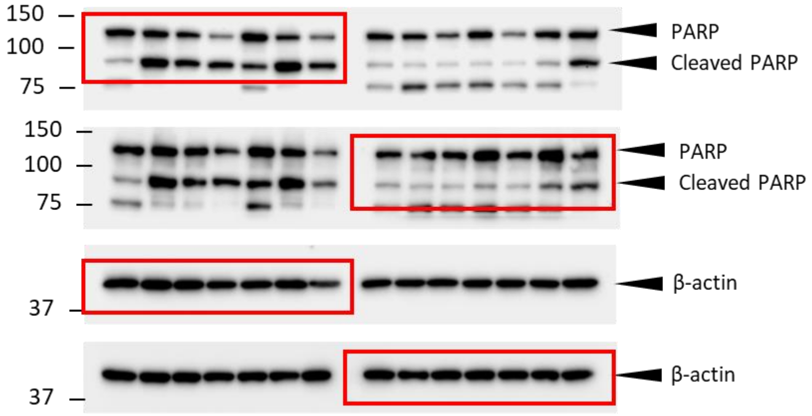

Supplementary Figure 73. Unprocessed and uncropped scans of original blots shown in Supplementary Figure 2C.

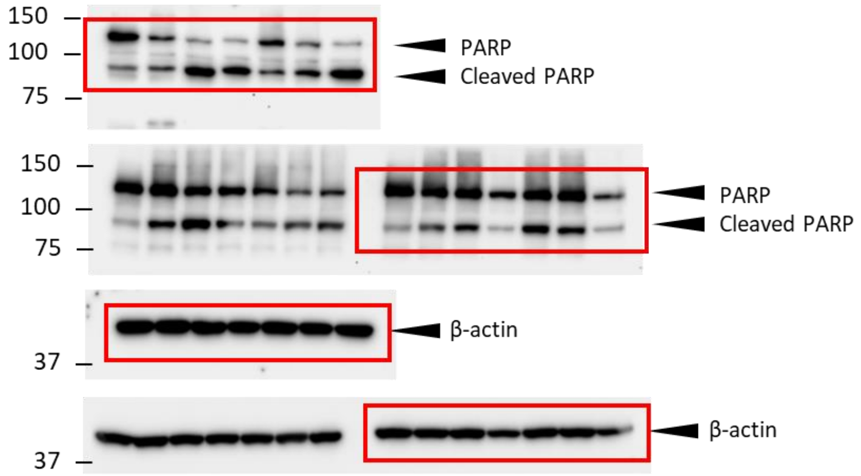

Supplementary Figure 74. Unprocessed and uncropped scans of original blots shown in Supplementary Figure 2D.

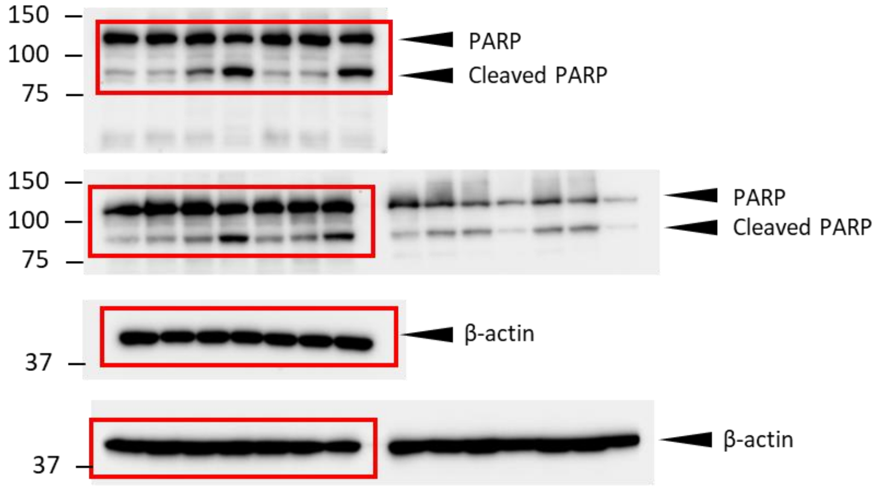

Supplementary Figure 75. Unprocessed and uncropped scans of original blots shown in Supplementary Figure 2E.

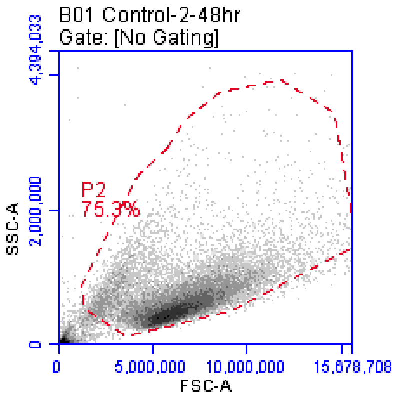

Supplementary Figure 76. Representative gating strategy of flow cytometry experiments in Supplementary Figure 3.

Supplementary Tables

Supplementary Table 1 *In vivo* anti-cancer effects of *Isodon* diterpenoids

	(–)-**1**	(±)-**1**	(±)-**2**	(±)-**3**	Cisplatin
Decrease in tumor volume	28.7% (*P* = 0.0426)	42.1% (*P* = 0.0224)	71.0% (*P* = 0.00001)	15.8% (NS)	34.1% (*P* = 0.0159)
Decrease in tumor weight	35.2% (*P* = 0.0348)	47.9% (*P* = 0.0078)	69.4% (*P* = 0.0003)	11.8% (NS)	37.8% (*P* = 0.0141)

NS: not significant, $*P < 0.05$, $**P < 0.01$, $***P < 0.001$ compared to control.

Supplementary Methods

Characterization data of all compounds

(1) Compound (±)-**4**.

^{1}H NMR (300 MHz, $CDCl_3$) δ ppm = 7.23 (d, *J* = 8.4 Hz, 2H), 6.85 (d, *J* = 8.4 Hz, 2H), 5.79-5.72 (m, 1H), 5.26-5.23 (m, 2H), 4.52 (d, *J* = 11.1 Hz, 2H), 4.27 (d, *J* = 11.1 Hz, 2H), 3.97-3.94 (m, 1H), 3.80 (s, 3H), 3.70-3.66 (m, 2H), 3.02 (s, 1H), 1.86-1.81 (m, 1H), 1.80-1.70 (m, 1H); ^{13}C NMR (76 MHz, $CDCl_3$) δ = 159.3, 138.3, 130.3, 129.4, 117.2, 113.9, 79.5, 70.0, 60.6, 55.3, 37.8. IR (neat, cm^{-1}) 3415, 2947, 2875, 2838, 1616, 1519, 1254, 1181, 1036, 818. HRMS (ESI/$[M+Na]^+$) calcd. for $C_{13}H_{18}O_3Na$: 245.1154, found 245.1155.

(2) Compound **5**

^{1}H NMR (500 MHz, $CDCl_3$) δ ppm = 3.76 (dd, *J* = 10.4, 2.8 Hz, 6H), 3.40 (qd, *J* = 10.4, 4.0 Hz, 1H), 2.80-2.90 (m, 1H), 2.55-2.65 (m, 1H), 2.37 (s, 3H), 1.98 (t, *J* = 2.4, Hz, 1H); ^{13}C NMR (126 MHz, $CDCl_3$) δ = 201.4 (d, *J* = 6.3 Hz), 80.5 (d, *J* = 25.0 Hz), 69.7 (d, *J* = 2.5 Hz), 53.2 (dd, *J* = 40.0, 8.3 Hz), 52.1 (d, *J* = 31.3 Hz), 31.2, 16.0 (d, *J* = 3.7 Hz); IR (neat, cm^{-1}) : 3476, 3290, 2963, 1721, 1254, 1044, 830. HRMS (ESI/$[M+Na]^+$) calcd. for $C_8H_{13}O_4PNa$: 227.0449, found 227.0456.

(3) Compound (±)-**6**

^{1}H NMR (300 MHz, $CDCl_3$) δ ppm = 7.24 (d, *J* = 8.4 Hz, 2H), 6.88 (d, *J* = 8.4 Hz, 2H), 6.76 (t, *J* = 7.2 Hz, 1H), 5.74-5.86 (m, 1H), 5.27-5.33 (m, 2H), 4.57 (d, *J* = 11.7 Hz, 1H), 4.31 (d, *J* = 11.7 Hz, 1H), 3.93 (dd, *J* = 13.2, 7.2 Hz, 1H), 3.80 (s, 3H), 3.20 (dd, *J* = 7.2, 2.7 Hz, 2H), 2.63 (dd, *J* = 12.0, 6.6 Hz, 2H), 2.30 (s, 3H), 1.88 (t, *J* = 2.7 Hz, 1H); ^{13}C NMR (75 MHz, $CDCl_3$) δ = 197.5, 159.2, 142.0, 138.2, 137.9, 130.3, 129.5, 118.1, 113.8, 81.4, 78.2, 69.8, 67.9, 55.3, 35.3, 25.4, 14.8. IR (neat, cm^{-1}) 3292, 3074, 3000, 2958, 2939, 2913, 2858, 2842, 1668, 1610, 1513, 1465, 1426, 1304, 1249, 1069, 1036, 824, 640. HRMS (ESI/$[M+Na]^+$) calcd. for $C_{19}H_{22}O_3Na$: 321.3718, found 321.3717.

(4) Compound **7**

^{1}H NMR (300 MHz, $CDCl_3$) δ ppm = 9.69 (s, 1H), 7.40 (s, 1H), 2.48 (dd, *J* = 7.5, 6.0 Hz, 2H), 1.85 (td, *J* = 7.8, 0.6 Hz, 2H), 1.19 (s, 6H); ^{13}C NMR (76 MHz, $CDCl_3$) δ =197.2, 189.4, 165.4, 131.5, 34.9, 34.4, 33.5, 27.0. IR (neat, cm^{-1}): 3645, 2963, 2927, 2867, 1733, 1685, 1608, 1346, 1177. HRMS (ESI/$[M+Na]^+$) calcd. for $C_9H_{12}O_2Na$: 175.0735, found 175.0739.

(5) Compound (±)-**9** (high R_f)

^{1}H NMR (300 MHz, pyridine-d_5) δ ppm = 7.53 (d, *J* = 8.4 Hz, 2H), 7.04 (d, *J* = 9.2 Hz, 2H), 5.81-5.90 (m, 1H), 5.44 (d, *J* = 15.9 Hz, 1H), 5.23 (dd, *J* = 10.2, 1.5 Hz, 1H), 4.56-4.78 (m, 4H), 4.20 (d, *J* = 9.3 Hz, 1H), 3.87 (dd, *J* = 17.1, 2.4 Hz, 1H), 3.81 (d, *J* = 10.5 Hz, 1H), 3.69 (s, 3H), 3.42 (d, *J* = 5.1 Hz, 1H), 2.85-2.98 (m, 4H), 2.39 (d, *J* = 6.6 Hz, 1H), 2.00-2.20 (m, 2H), 1.77 (d, *J* = 10.5 Hz, 1H), 1.54 (dd, *J* = 13.5, 1.5 Hz, 1H), 1.40-1.50 (m, 2H), 1.21 (s, 3H), 1.13 (m, 21H), 1.04 (s, 3H); ^{13}C NMR (76 MHz, pyridine-d_5) δ = 159.6, 145.2, 138.9, 130.4, 130.1, 116.4, 113.88, 111.2, 83.5, 83.1, 73.9, 71.2, 69.7, 59.9, 54.8, 44.8, 41.60, 41.4, 35.4, 33.8, 33.7, 33.3, 28.4, 28.0, 22.9, 17.9, 17.8, 16.2, 13.0; IR (neat, cm^{-1}) 3428, 2945, 2900, 2868, 1739, 1681, 1619, 1520, 1465, 1246, 1217, 1169, 1088, 1043, 988, 885, 811, 673. HRMS (ESI/[M+Na]$^+$) calcd. for $C_{37}H_{58}O_5SiNa$: 633.3951, found 633.3959.

(6) Compound (±)-**9** (low R_f)

^{1}H NMR (500 MHz, CD_3CN) δ ppm = 7.31 (d, *J* = 8.5 Hz, 2H), 6.92 (d, *J* = 8.5 Hz, 2H), 5.80-5.88 (m, 1H), 5.34 (dd, *J* = 17.5, 2.0 Hz, 1H), 5.29 (dd, *J* = 10.5, 2.0 Hz, 1H), 4.54 (d, *J* = 12.0 Hz, 1H), 4.28 (d, *J* = 12.0 Hz, 1H), 4.14 (dd, *J* = 12.0, 6.0 Hz, 1H), 4.01 (d, *J* = 8.0 Hz, 1H), 3.82 (s, 3H), 3.75 (d, *J* = 8.0 Hz, 1H), 3.30-3.40 (m, 2 H), 2.91 (d, *J* = 3.5, Hz, 1H), 2.77-2.86 (m, 3H), 2.35-2.45 (m, 2H), 2.07 (t, *J* = 2.5 Hz, 1H), 1.94 (d, *J* = 17.5 Hz, 1H), 1.64-1.71 (m, 1H), 1.61 (d, *J* = 7.0 Hz, 1H), 1.42-1.50 (m, 2H), 1.30-1.41 (m, 2H), 1.15-1.25 (m, 21H), 0.90 (s, 3H), 0.87 (s, 3H); ^{13}C NMR (126 MHz, CD_3CN) δ = 159.2, 145.0, 140.1, 131.2, 129.8, 116.6, 113.7, 114.0, 84.5, 80.7, 73.9, 69.4, 67.8, 61.3, 54.9, 44.3, 42.3, 40.6, 34.4, 34.3, 33.6, 32.9, 27.6, 27.5, 22.3, 17.7, 17.6, 16.5, 13.2. IR (neat, cm^{-1}) 3437, 2946, 2865, 1675, 1613, 1517, 1465, 1365, 1249, 1204, 1175, 1036, 885, 824, 669, 618. HRMS (ESI/[M+Na]$^+$) calcd. for $C_{37}H_{58}O_5SiNa$: 633.3951, found 633.3963.

(7) Compound (±)-**10**

^{1}H NMR (300 MHz, $CDCl_3$) δ ppm = 6.44 (dd, *J* = 17.5, 10.5 Hz, 1H), 6.20 (dd, *J* = 17.4, 1.5 Hz, 1H), 5.64 (dd, *J* = 13.5, 1.5, 1H), 3.89 (dd, *J* = 12.0, 3.9, 1H), 3.82 (d, *J* = 9.6, 1H), 3.71 (d, *J* = 7.5, 1H), 3.62 (d, *J* = 18.0, 1H), 3.43 (d, *J* = 9.6, 1H), 2.95 (d, *J* = 17.1, 1H), 2.79 (dd, *J* = 18.0, 8.7 Hz, 1H), 2.63 (d, *J* = 17.1Hz, 1H), 2.19-2.35 (m, 1H), 2.01 (d, *J* = 12.3 Hz, 1H), 1.76-2.02 (m, 2H), 1.73 (t, *J* = 2.4 Hz, 1H), 1.51-1.56 (m, 1H), 1.40 (dt, *J* = 13.5, 3.3 Hz, 1H), 1.15-1.33 (m, 2H), 0.98-1.19 (m, 21H), 0.83-0.98 (m, 15H), 0.78-0.81 (m, 9H), 0.07 (s, 3H), 0.05 (s, 3H), 0.03 (s, 3H), 0.02 (s, 3H). ^{13}C NMR (76 MHz, $CDCl_3$) δ = 199.8, 144.7, 136.4, 126.2, 110.8, 84.0, 75.2, 67.4, 60.2, 44.1, 41.9, 41.3, 40.5, 33.5, 33.4, 32.3, 27.8, 27.4, 25.8, 25.7, 22.6, 18.1,

18.0, 17.9, 16.4, 13.2, -4.6, -4.9, -5.0, -6.0. IR (neat, cm^{-1}) 3313, 2953, 2860, 1683, 1615, 1469, 1389, 1364, 1252, 1215, 1084, 1010, 883, 836, 780, 737, 681. HRMS (ESI/$[M+Na]^+$) calcd. for $C_{41}H_{76}O_4Si_3Na$:739.4949, found 739.4967.

(8) Compound (±)-**11**

^{1}H NMR (500 MHz, $CDCl_3$) δ ppm = 5.16 (s, 1H), 4.98 (s, 1H), 3.91 (d, *J* = 10.0 Hz, 1H), 3.83 (dd, *J* = 12.0, 4.0 Hz, 1H), 3.72 (d, *J* = 10.0 Hz, 1H), 3.27 (t, *J* = 1.5 Hz, 1H), 3.19 (dt, *J* = 6.0, 3.0 Hz, 1H), 2.82-2.89 (m, 3H), 2.62 (dd, *J* = 18.5, 8.0 Hz, 1H), 2.51 (dd, *J* = 18.5, 2.5 Hz, 1H), 2.39 (d, *J* = 16.5 Hz, 1H), 2.14 (d, *J* = 4.0 Hz, 2H), 2.06 (dd, *J* = 8.0, 2.5 Hz, 1H), 1.70-1.74 (m, 1H), 1.57-1.60 (m, 1H), 1.46 (dt, *J* = 8.5, 3.5 Hz, 1H), 1.30-1.39 (m, 1H), 0.90-0.98 (m, 18H), 0.08 (s, 3H), 0.07 (s, 3H), 0.07 (s, 3H), 0.06 (s, 3H). ^{13}C NMR (126 MHz, $CDCl_3$) δ = 214.6, 209.2, 145.7, 109.7, 75.4, 61.4, 58.3, 53.7, 47.0, 46.6, 44.0, 40.0, 39.4, 37.9, 37.5, 33.8, 33.0, 27.5, 26.0, 25.9, 25.6, 24.3, 18.1, 18.0, -4.3, -4.8, -5.1, -5.7. IR (neat, cm^{-1}) 2953, 2867, 1708, 1475, 1258, 1097, 836, 774, 672. HRMS (ESI/$[M+Na]^+$) calcd. for $C_{32}H_{56}O_4Na$: 583.3615, found 583.3618.

(9) Compound (±)-**12**

^{1}H NMR (500 MHz, $CDCl_3$) δ ppm = 5.23 (s, 1H), 5.00 (s, 1H), 4.44 (d, *J* = 9.5 Hz, 1H), 3.44 (dd, *J* = 9.0, 2.0 Hz, 1H), 3.30 (dd, *J* = 12.0, 3.0 Hz, 1H), 3.23 (d, *J* = 4.0 Hz, 1H), 2.85 (dd, *J* = 17.5, 2.0 Hz, 1H), 2.69 (dt, *J* = 16.0, 2.5 Hz, 1H), 2.49 (dd, *J* = 17.5, 6.0 Hz, 1H), 2.33-2.39 (m, 1H), 2.07 (d, *J* = 16.0 Hz, 1H), 1.89-2.05 (m, 3H), 1.77 (dd, *J* = 7.5, 5.5 Hz, 1H), 1.66 (dd, *J* = 11.5, 7.5 Hz, 1H), 1.44-1.60 (m, 3H), 1.25-1.35 (m, 1H), 1.29 (s, 3H), 0.86 (s, 3H), 0.85 (s, 9H), 0.01 (s, 3H), 0.00 (s, 3H). ^{13}C NMR (125 MHz, $CDCl_3$) δ = 210.9, 145.2, 111.4, 96.7, 79.6, 72.5, 56.4, 49.3, 47.7, 42.7, 40.0, 40.0, 38.4, 35.7, 33.5, 33.4, 32.2, 30.7, 28.3, 25.8, 23.1, 17.9, -4.4, -5.1. IR (neat, cm^{-1}) 3411, 2958, 2935, 2903, 2865, 1713, 1700, 1652, 1468, 1320, 1256, 1140, 1123, 1101, 1075, 885, 843, 772, 666. HRMS (ESI/$[M+Na]^+$) calcd. for $C_{26}H_{42}O_4SiNa$: 469.2750, found 469.2753.

(10) Compound (±)-**13**

^{1}H NMR (400 MHz, $CDCl_3$) δ ppm = 5.10 (s, 1H), 5.02 (s, 1H), 4.67-4.72 (m, 1H), 4.05 (dd, *J* = 10.4, 3.2 Hz, 1H), 3.99 (d, *J* = 9.6 Hz, 1H), 3.91 (d, *J* = 9.6 Hz, 1H) 3.35-3.45 (m, 2H), 3.06 (s, 1H), 2.97 (s, 3H), 2.84 (d, *J* = 9.6 Hz, 1H), 2.50-2.52 (m, 2H), 2.00-2.05 (m, 2H), 1.92 (d, *J* = 12.0 Hz, 1H), 1.60-1.75 (m, 3H), 1.52-1.57 (m, 1H), 1.40-1.46 (m, 1H), 1.22-1.35 (m, 1H), 0.90-0.98 (m, 21H), 0.78 (s, 3H), 0.04-0.15 (m, 12H). ^{13}C NMR (101 MHz, $CDCl_3$) δ 213.8, 146.2, 109.0, 81.5, 74.6, 60.8, 55.1, 47.3, 47.1, 43.7, 41.8, 41.7, 41.6, 39.9, 38.8, 37.4, 34.0, 32.9, 27.1, 25.8,

25.4, 24.8, 18.0, 17.8, -4.9, -5.0, -5.0, -5.7. IR (neat, cm^{-1}) 2941, 2860, 1702, 1475, 1357, 1258, 1184, 1097, 939, 836, 777, 669, 532. HRMS (ESI/$[M+Na]^+$) calcd. for $C_{33}H_{60}O_6SSi_2Na$: 663.3547, found 663.3545.

(11) Compound (±)-**18**

^{1}H NMR (500 MHz, $CDCl_3$) δ ppm = 4.80 (s, 1H), 4.72 (s, 1H), 4.70 (d, *J* = 6.5 Hz, 1H), 4.05 (dd, *J* = 12.5, 3.0 Hz, 1H), 3.81 (q, *J* = 8.5 Hz, 2H), 2.75 (d, *J* = 16.5 Hz, 1H), 2.57-2.61 (m, 1H), 2.50-2.53 (m, 1H), 2.37 (dd, *J* = 8.0, 4.5 Hz, 1H), 2.03-2.06 (m, 2H), 1.80-1.83 (m, 1H), 1.68-1.77 (m, 2H), 1.57-1.63 (m, 1H), 1.44-1.51 (m, 3H), 1.35-1.44 (m, 1H), 1.27-1.35 (m, 1H), 0.81-0.99 (m, 24H), 0.22 (s, 9H), 0.07 (m, 12H). ^{13}C NMR (126 MHz, $CDCl_3$) δ = 157.4, 153.5, 102.2, 102.2, 74.8, 62.1, 47.3, 47.1, 44.7, 44.6, 41.6, 40.8, 39.8, 36.4, 34.0, 33.7, 32.7, 27.4, 26.1, 25.9, 25.0, 18.4, 18.0, 0.6, -4.4, -4.8, -5.0, -5.3. IR (neat, cm^{-1}) 2961, 2923, 2868, 2852, 1745, 1661, 1639, 1465, 1378, 1252, 1101, 1078, 853, 776. HRMS (ESI/$[M+Na]^+$) calcd. for $C_{35}H_{66}O_3Si_3Na$: 641.4217, found 641.4225.

(12) Compound (±)-**14**

^{1}H NMR (400 MHz, $CDCl_3$) δ ppm = 4.82 (s, 1H), 4.79 (s, 1H), 4.37 (dd, *J* = 9.6, 6.4 Hz, 1H), 4.29 (d, *J* = 1.2, 1H), 3.81 (d, *J* = 9.2 Hz, 1H), 3.59 (d, *J* = 5.2 Hz, 1H), 3.50 (d, *J* = 9.6 Hz, 1H), 2.83 (s, 1H), 2.77 (d, *J* = 16.8 Hz, 1H), 2.65-2.70 (m, 2H), 2.45 (d, *J* = 8.0 Hz, 1H), 2.19 (d, *J* = 16.0 Hz, 1H), 1.78-1.94 (m, 2H), 1.66-1.78 (m, 2H), 1.50-1.52 (m, 1H), 1.35-1.42 (m, 3H), 1.06 (s, 3H), 0.88-0.95 (m, 21H), 0.10 (s, 3H), 0.08 (s, 3H), 0.07 (s, 3H), 0.06 (s, 3H). ^{13}C NMR (101 MHz, $CDCl_3$) δ = 212.3, 153.8, 103.4, 77.0, 69.8, 63.6, 57.2, 52.1, 50.4, 44.1, 42.5, 40.8, 34.8, 33.7, 33.6, 32.0, 32.0, 28.9, 26.9, 25.9, 25.7, 19.8, 18.1, 17.8, -4.5, -5.2, -5.4, -5.7. IR (neat, cm^{-1}) 3486, 2941, 1699, 1472, 1255, 1097, 839, 777, 669. HRMS (ESI/$[M+Na]^+$) calcd. for $C_{32}H_{58}O_4Si_2Na$: 585.3771, found 585.3773.

(13) Compound (±)-**15**

^{1}H NMR (400 MHz, $CDCl_3$) δ ppm = 4.98 (d, *J* = 1.2 Hz, 1H), 4.77 (s, 1H), 4.11-4.30 (br, 1H), 4.10 (d, *J* = 10.0 Hz, 1H), 3.99 (dd, *J* = 9.6, 2.0 Hz, 1H), 3.63 (d, *J* = 2.0 Hz, 1H), 2.70 (dd, *J* = 8.8, 4.8 Hz, 1H), 2.30-2.41 (m, 2H), 2.18-2.27 (m, 1H), 1.75-1.95 (m, 3H), 1.65-1.71 (m, 2H), 1.55-1.62 (m, 3H), 1.45-1.55 (m, 1H), 1.39 (s, 3H), 1.21-1.28 (m, 1H), 1.14 (s, 3H); ^{13}C NMR (101 MHz, $CDCl_3$) δ = 207.56, 157.0, 106.4, 93.7, 65.0, 64.9, 56.8, 46.9, 45.8, 44.1, 42.7, 38.0, 34.4, 33.9, 33.7, 31.1, 28.3, 26.3, 22.0, 15.1. IR (neat, cm^{-1}) 3469, 2958, 2939, 2865, 1729, 1665, 1500, 1458, 1388, 1365, 1162, 1111, 1078, 1056, 972, 879. HRMS (ESI/$[M+Na]^+$) calcd. for $C_{20}H_{28}O_4Na$: 355.1885, found 355.1890.

(14) Compound (±)-**16**

^{1}H NMR (400 MHz, CD_3OD) δ ppm = 5.18 (s, 1H), 5.17 (s, 1H), 4.06-4.12 (m, 2H), 3.65 (s, 1H), 3.54 (s, 1H), 2.74 (t, *J* = 4.4 Hz, 1H), 2.32 (s, 1H), 2.15-2.24 (m, 1H), 2.07 (dd, *J* = 9.6, 5.2 Hz, 1H), 1.75-1.85 (m, 3H), 1.65-1.74 (m, 2H), 1.52-1.62 (m, 1H), 1.43-1.53 (m, 2H), 1.28 (s, 3H), 1.15-1.22 (m, 1H), 1.07 (s, 3H); ^{13}C NMR (101 MHz, CD_3OD) δ = 208.0, 159.9, 110.7, 94.7, 80.7, 64.0, 63.9, 56.9, 48.5, 43.7, 42.2, 35.8, 34.0, 33.5, 33.1, 31.6, 25.6, 24.4, 21.3, 14.2. IR (neat, cm^{-1}) 3411, 2935, 2868, 1732, 1497, 1458, 1420, 1365, 1275, 1146, 1065, 991, 908. HRMS (ESI/$[M+Na]^+$) calcd. for $C_{20}H_{28}O_5Na$: 371.1834, found 371.1837.

(15) (±)-15-*epi*-Enmelol (**17**)

^{1}H NMR (500 MHz, pyridine-d_5) δ ppm = 7.94 (s, 1H), 6.97 (d, *J* = 3.5 Hz, 1H), 6.31 (s, 1H), 5.73 (d, *J* = 3.0 Hz, 1H), 5.32 (s, 1H), 5.16 (s, 1H), 5.09 (s, 1H), 5.03 (s, 1H), 4.39 (s, 1H), 4.16 (s, 2H), 3.70 (s, 1H), 2.65-2.76 (m, 2H), 1.99-2.34 (m, 6H), 1.72-1.85 (m, 3H), 1.52-1.62 (m, 1H), 1.34 (s, 3H), 1.20-1.30 (m, 1H), 1.24 (s, 3H). ^{13}C NMR (126 MHz, pyridine-d_5) δ = 163.2, 109.6, 98.3, 82.0, 74.6, 65.2, 65.1, 52.8, 50.8, 44.9, 41.0, 36.0, 34.3, 33.8, 33.5, 32.6, 27.8, 27.5, 23.2, 15.2. IR (neat, cm^{-1}) 3313, 2972, 2886, 1723, 1190, 1063, 963. HRMS (ESI/$[M+Na]^+$) calcd. for $C_{20}H_{30}O_5Na$: 373.1991. found 373.1998.

(16) (±)-Xerophilusin I (**3**)

^{1}H NMR (400 MHz, pyridine-d_5) δ ppm = 7.05-7.25 (br, 1H), 5.96 (s, 1H), 5.88 (s, 1H), 5.26 (s, 1H), 4.35 (d, *J* = 4.0 Hz, 1H), 4.13 (dd, *J* = 8.0, 2.5 Hz, 2H), 3.68 (s, 1H), 2.93 (dd, *J* = 12.0, 4.0 Hz, 1H), 2,48-2.61 (m, 2H), 2.40 (d, *J* = 12.0 Hz, 2H), 2.10-2.29 (m, 4H), 1.87-1.94 (m, 1H), 1.69-1.85 (m, 2H), 1.32-1.52 (m, 1H), 1.31 (s, 3H), 1.24 (d, *J* = 13.2 Hz, 2H), 1.16 (s, 3H); ^{13}C NMR (101 MHz, pyridine-d_5) δ = 212.1, 155.1, 117.3, 97.4, 75.6, 67.0, 65.7, 60.9, 57.4, 47.0, 42.3, 36.2, 35.4, 35.2, 34.7, 30.9, 28.6, 28.0, 24.1, 17.3. IR (neat, cm^{-1}) 3453, 3360, 3233, 2952, 2935, 2923, 2865, 1710, 1642, 1636, 1452, 1420, 1336, 1281, 1201, 1078, 1014, 946. HRMS (ESI/$[M+Na]^+$) calcd. for $C_{20}H_{28}O_5Na$: 371.1834, found 371.1842.

(17) (±)-Neolaxiflorin L (**2**)

^{1}H NMR (500 MHz, pyridine-d_5) δ ppm = 8.91-9.12 (br, 1H), 6.30 (d, *J* = 16.5 Hz, 1H), 6.06 (s, 1H), 5.02 (s, 1H), 4.59 (d, *J* = 10.0 Hz, 1H), 4.17-4.22 (m, 2H), 2.89 (dd, *J* = 9.0, 4.0 Hz, 1H), 2,65-2.71 (m, 1H), 2.42-2.53 (m, 2H), 2.30-2.36 (m, 1H), 2.25-2.29 (m, 2H), 1.96-2.13 (m, 2H), 1.82-1.88 (m, 1H), 1.56-1.61(m, 1H), 1.29-1.34 (m, 2H), 1.25 (s, 3H), 0.91 (s, 3H); ^{13}C NMR (126 MHz, pyridine-d_5) δ = 212.3,

208.6, 153.3, 117.8, 96.5, 73.6, 64.7, 61.5, 59.8, 49.0, 47.6, 38.7, 35.7, 34.6, 32.8, 30.7, 29.7, 25.6, 23.2, 18.8. IR (neat, cm^{-1}) 3460, 2958, 2923, 2852, 1742, 1700, 1636, 1458, 1085, 1059. HRMS (ESI/$[M+Na]^+$) calcd. for $C_{20}H_{26}O_5Na$: 369.1678, found 369.1687.

(18) (±)-Eriocalyxin B (**1**)
^{1}H NMR (400 MHz, pyridine-d_5) δ ppm = 6.73 (d, *J* = 10.4 Hz, 1H), 6.46 (d, *J* = 11.2 Hz, 1H), 6.04 (s, 1H), 6.03 (d, *J* = 10.4 Hz, 1H), 5.36 (s, 1H), 4.56 (dd, *J* = 10.0, 1.2 Hz, 1H), 4.35 (dd, *J* = 6.0, 6.0 Hz, 1H), 4.17 (dd, *J* = 10.0, 1.6 Hz, 1H), 2.89 (d, *J* = 9.2 Hz, 1H), 2.49-2.55 (m, 2H), 2.12-2.35 (m, 3H), 1.96-2.00 (m, 1H), 1.58 (dd, *J* = 10.0, 2.0 Hz, 1H), 1.37 (s, 3H), 1.28-1.47 (m, 1H), 1.11 (s, 3H); ^{13}C NMR (101 MHz, pyridine-d_5) δ = 210.0, 198.0, 161.6, 154.3, 128.1, 118.5, 97.3, 74.2, 66.2, 61.0, 59.1, 48.9, 47.3, 36.7, 35.4, 30.8, 30.6, 26.5, 25.2, 20.3. IR (neat, cm^{-1}) 3340, 2980, 2949, 2878, 2851, 1710, 1664, 1647, 1073, 1059, 951. HRMS (ESI/$[M+Na]^+$) calcd. for $C_{20}H_{24}O_5Na$: 367.1521, found 367.1522.

(19) (–)-Eriocalyxin B (**1**)
^{1}H NMR (300 MHz, pyridine-d_5) δ ppm = 6.72 (d, *J* = 7.8 Hz, 1H), 6.46 (d, *J* = 8.4 Hz, 1H), 6.02 (d, *J* = 9.9Hz, 2H), 5.35 (s, 1H), 4.56 (d, *J* = 9.9 Hz, 1H), 4.36 (t, *J* = 4.5 Hz, 1H), 4.17 (d, *J* = 9.9 Hz, 1H), 2.89 (d, *J* = 9.0 Hz, 1H), 2.50 (s, 2H), 2.12-2.24 (m, 3H), 1.94-1.98 (m, 1H), 1.60 (m, 1H), 1.41 (s, 3H), 1.26-1.30 (m, 1H), 1.10 (s, 3H); ^{13}C NMR (76 MHz, pyridine-d_5) δ = 210.4, 198.5, 162.0, 154.6, 128.5, 119.0, 97.7, 74.6, 66.6, 61.4, 59.5, 49.3, 47.7, 37.1, 35.8, 31.2, 30.0, 26.9, 25.6, 20.7. IR (neat, cm^{-1}) 3340, 2982, 2949, 2878, 2853, 1710, 1664, 1647, 1070, 1059, 950. HRMS (ESI/$[M+Na]^+$) calcd. for $C_{20}H_{24}O_5Na$: 367.1521, found 367.1520.

(20) (+)-Eriocalyxin B (**1**)
^{1}H NMR (300 MHz, pyridine-d_5) δ ppm = 9.21 (s, 1H), 6.71 (d, *J* = 9.9 Hz, 1H), 6.45 (d, *J* = 11.4 Hz, 1H), 6.01 (d, 2H), 6.03 (d, *J* = 9.9Hz, 2H), 5.34 (s, 1H), 4.54 (d, *J* = 3.6 Hz, 1H), 4.31-4.37 (m, 1H), 4.15 (d, *J* = 3.6 Hz, 1H), 2.87 (d, *J* = 9.0 Hz, 1H), 2.49 (s, 2H), 2.10-2.24 (m, 3H), 1.93-1.98 (m, 1H), 1.61 (dd, *J* = 7.5, 1.5 Hz, 1H), 1.52 (s, 3H), 1.31-1.40 (m, 1H), 1.26 (s, 3H); ^{13}C NMR (126 MHz, pyridine-d_5) δ = 209.0, 197.0, 160.7, 153.4, 127.2, 117.6, 96.4, 73.3, 65.2, 60.1, 58.2, 48.1, 46.4, 35.8, 34.5, 29.9, 29.7, 25.6, 24.2, 19.3. IR (neat, cm^{-1}) 3342, 2980, 2949, 2879, 2851, 1712, 1664, 1647, 1075, 1059, 951. HRMS (ESI/$[M+Na]^+$) calcd. for $C_{20}H_{24}O_5Na$: 367.1521, found 367.1523
